# NMR “Finger Prints” of N-Heterocyclic Carbenes, DFT Analysis: Scopes and Limitations

**DOI:** 10.3390/molecules28237729

**Published:** 2023-11-23

**Authors:** Svetlana A. Kondrashova, Shamil K. Latypov

**Affiliations:** Arbuzov Institute of Organic and Physical Chemistry, FRC Kazan Scientific Center of RAS, 420088 Kazan, Russia; kondrashovamail@gmail.com

**Keywords:** nitrogen heterocycles, carbenes, DFT calculations, NMR spectroscopy, selenium, phosphorus, ligand properties

## Abstract

The scopes and limitations of theoretical methods for estimating various NMR metrics of NHCs are considered on a wide range of model NHCs and their derivatives, using a number of computational approximations. On the whole, the most reliable, from the point of view of predictability and insusceptibility to additional effects, are ^31^P/^13^C NMR shifts of NHC–phosphinidene adducts and ^13^C CSs of carbenes themselves. The method based on the analysis of ^77^Se CS NHC–selenoureas has some limitations since the observed NMR parameters can also be modulated by exchange effects due to their formation with non-classical hydrogen bonds. As for HEP, since the delicate balance of electron distribution between Pd and two carbon centers can nonlinearly affect relativistic spin–orbit effects, the accuracy of the estimation of this metric may still be limited. ^13^C CSs of NHC–azolium salts do not seem to be reliable, since the observed values are strongly influenced by the effects of the exchange between different forms with counterions, which are difficult to estimate correctly.

## 1. Introduction

Since the discovery of N-heterocyclic carbenes (NHC), they have been widely used in chemical science [1,2,3]. Due to their unique electronic structure, NHCs form stable complexes with transition metals (TM), which are interesting from the point of view of catalysis [4,5,6,7,8,9].

For the rational design of such systems, information is needed on the electronic properties of such systems and criteria for qualitative/quantitative assessment of stereoelectronic characteristics. A number of methods have been proposed for this purpose [10,11,12]. For example, the Tolman electronic parameter is based on measuring the IR frequencies of the CO vibrations in [(NHC)Ni(CO)_3_] or [*cis*-(CO)_2_-(NHC)MCl] (M = Rh or Ir) complexes [11,13,14]. However, the accuracy of the method is not sufficient, since the range of changes is small within a relatively large line width. Moreover, the use of toxic carbon monoxide gas is often still required in the preparation of the respective complex probes.

In principle, electronic properties of NHCs can be evaluated using their ligand electrochemical parameter or redox potentials [11]. However, only complexes that exhibit a reversible or quasireversible redox process can be investigated. Moreover, since the NHCs with a redox-active groups are considered non-innocent, they cannot be evaluated properly using this method.

A number of NMR-based methods have emerged in recent years. For example, ^13^C NMR spectroscopy of NHC complexes [PdBr_2_(*^i^*Pr_2_-bimy)*L*] was provided to measure the donor ability of the *trans*-ligand *L* [11,15,16,17]. Later, the use of ^77^Se NMR spectroscopy on NHC–selenium adducts (selenoureas) was suggested to characterize NHCs [11,18,19,20]. Along the same line, the use of ^31^P NMR spectroscopy on NHC–phosphinidene adducts was proposed [11,19,20,21]. Most recently, the used of ^1^*J*(C–H) coupling constants in azolium salts was suggested as a measure for the σ-donating ability of the respective NHC [11].

The advantage of the NMR-based methods is their high sensitivity to structural modifications. A large range of the NMR chemical shift (CS) changes with a small line width makes it easy to differentiate and compare these parameters. From a practical point of view, it is also important that samples are relatively easy to prepare and carry out appropriate measurements. Moreover, some intuitive correlations have been proposed between the NHC properties and the NMR parameters of the corresponding derivatives [11].

On the other hand, in recent years there has been huge progress in methods of nonempirical estimation of NMR parameters. Particularly notable are advances in CS calculations of main group elements, such as ^13^C, ^15^N and ^31^P [22,23,24,25,26,27,28]. ^13^C and ^31^P CSs in nickel and palladium complexes have also been calculated, including taking into account relativistic effects [29,30,31,32,33]. As for heavier atoms such as ^77^Se, there are also examples of calculations, although fairly high levels of theory were used [34,35,36,37].

Therefore, in principle, there is a potential opportunity to predict the CS for new carbenes and their derivatives, and thus evaluate the electronic properties of new NHCs in NMR metrics. To our knowledge, there is only one example in the literature of calculating the CSs of the corresponding NHC adducts [20]. However, the accuracy of these calculations is not good enough. This may be due to the poor choice of the protocol for conducting such estimates. Specifically, the authors used a GGA functional that is unreliable, particularly for nuclei with large paramagnetic contribution in its shielding [30,38]. In practice, the molecular system may also be influenced by additional factors, which can lead to discrepancies between prediction and experiment. In this work, we will try to answer the question of whether it is possible to predict the NMR CSs in NHC derivatives and which of the NMR-based parameters is more reliable in terms of predictability.

## 2. Results and Discussion

First, we searched for NMR data available in the literature for NHCs and their derivatives in order to use them as models. In general, analysis of experimental data allows us to conclude that methods based on ^31^P NMR shifts of NHC–phosphinidene adducts, ^77^Se NMR shifts of NHC-derived selenoureas and ^13^C NMR of NHCs themselves are more sensitive to the structure of NHCs, since the range of changes in the corresponding CSs is large (ca. 190, 1200 and 150 ppm, for ^31^P-, ^77^Se- and ^13^C-based methods, respectively). For NHC–azolium salts and the Huynh’s Electronic Parameter (HEP) (^13^C NMR spectroscopy of NHC complexes [PdBr_2_(*^i^*Pr_2_-bimy)*L*]) the variation of ^13^C NMR shifts is significantly smaller (<25 ppm).

To start, we used the PBE0/6-311G(2d,2p)//PBE0/6-31+G(d)) combination for the calculation, which has proven itself well for ^13^C and ^31^P CSs calculations of organic compounds. For palladium complexes, ^13^C shift (HEP) calculations were carried out at a fully relativistic matrix Dirac–Kohn–Sham (mDKS) level of theory.

### 2.1. ^77^Se-NMR Shifts of NHC–Derived Selenoureas

#### 2.1.1. General Overview

**Figure 1:**
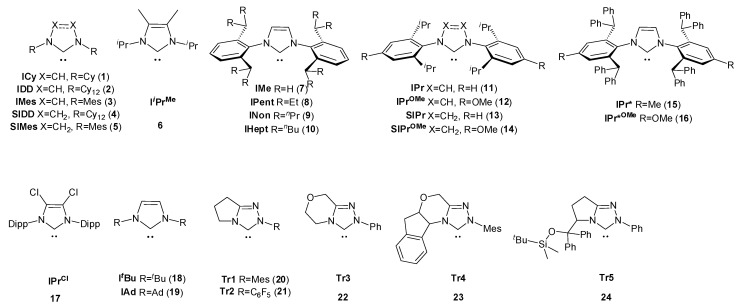
The main training set of carbene structures **1**–**24** used for ^77^Se shift calculations.

As the main training set at the first stage, the same diverse set (Figure 1) described in the ref. [20] was used, which contains 24 accurate experimental data in a low polarity solvent (CHCl_3_).

**Figure 2:**
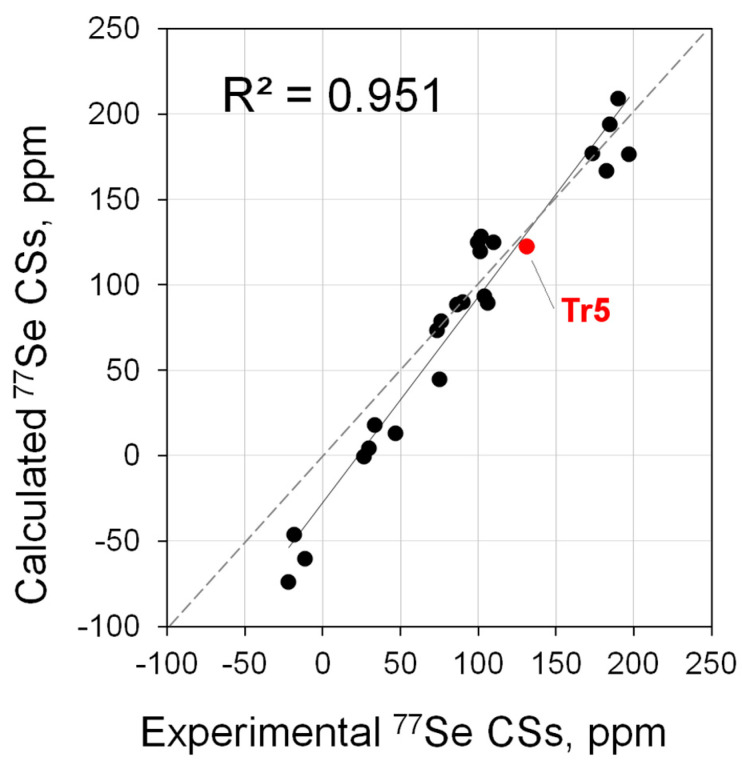
Correlation of calculated (PBE0/6-311G(2d,2p)//PBE0/6-31+G(d)) vs. experimental ^77^Se NMR shifts for the main set of carbene Se adducts (**1**·Se–**24**·Se).

In general, the correlation between the calculated and experimental ^77^Se shifts is much better than that obtained by the authors in ref. [20]. For example, when using the BP86/TZ2P//BP86/TZ2P combination, the correlation coefficient between calculation and experiment was *R*^2^ = 0.887 or 0.938 (without **Tr5** (**24**)), while in our case it was *R*^2^ = 0.951, taking into account all points (Figure 2, Table S1). It is noteworthy that even for **Tr5**, which seemed problematic and therefore the authors excluded it from the correlation analysis, the calculations fit the general dependence quite well. At the same time, a certain variation is observed, especially clearly visible for data in the low- and high-field regions of spectra (Figure 2).

A detailed analysis of systems with ^77^Se resonance in high fields (ICy (**1**), IDD (**2**), I^i^Pr^Me^ (**6**)) showed that such a variation in the correlation may be due to an equilibrium of the forms with and without an unusual non-classical hydrogen bond (NCHB) [39] between *^i^*Pr hydrogen atoms and the Se atom (e.g., for I^i^Pr^Me^·Se, see Figure S1). For these compounds, structures with such an NCHB correspond to a minimum and are only slightly lower in energy than the states without such a bond (Table S2). Taking into account that the ^77^Se CSs differ dramatically in these conformers, even small changes in the populations of the forms can lead to strong shifts in the average signal in the ^77^Se NMR spectrum. In principle, it is difficult to reliably determine the populations of such forms in solutions, which in turn makes it difficult to assess the contribution of each form to the average spectrum. This is most likely the main source of uncertainty in ^77^Se NMR shifts for systems in high fields. For systems in low fields, such unusual structural isomers stabilized by NCHBs have not been identified.

Next, an attempt was made to improve the quality of the calculation procedure by variation of parameters: the solvent effect, the influence of the basis set and the functional, and the influence of relativistic effects.

#### 2.1.2. Solvent Effects

**Figure 3:**
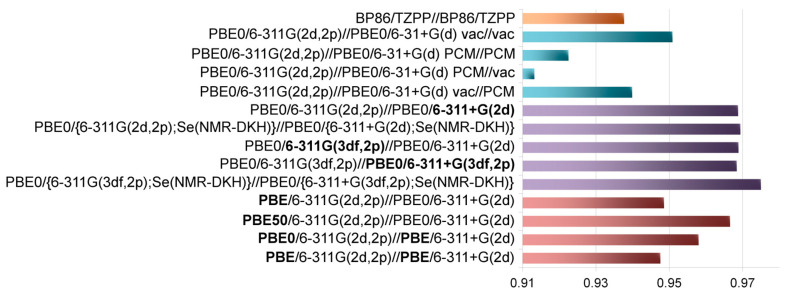
*R*^2^ dependence of ^77^Se NMR shifts for the main set of carbene Se adducts (**1**·Se–**24**·Se) on combinations used in calculations.

Solvent effects were taken into account within the framework of the polarization continuum model (PCM) [40]. Calculations were carried out for chloroform, in which the experimental data were obtained. First, the solvent was taken into account in both the geometry optimization and shielding calculations. The results were somewhat unexpected. Specifically, if usually when calculating NMR parameters when going from vacuum to solvent, an improvement or at least not a strong change is observed, then in this case, firstly, very strong changes in absolute value are predicted, and secondly, and this is very important, the correlation between calculated and experimental values significantly deteriorates (*R*^2^ = 0.923 vs. 0.951, Figure 3, Table S1).

To assess which of the stages—geometry optimization or shielding calculation—is most sensitive to the solvent effects, we carried out shielding calculations, taking into account the solvent, on geometry optimized for vacuum, and similar shielding calculations in vacuum, but for the geometry obtained taking into account the solvent effect. The influence of the solvent precisely at the shielding calculation stage leads to strong changes (*R*^2^ = 0.913, Figure 3, Table S1). At the same time, the influence of the solvent at the geometry optimization stage also leads to a worsening in the correlation (*R*^2^ = 0.940, Table S1), although not so drastically. Thus, for purely practical reasons, the calculations for vacuum look preferable so far.

#### 2.1.3. The Quality of the Basis Sets

Next, to improve the correlation of the calculation with the experiment, we augmented the basis sets both at the geometry optimization and at the shielding calculations, and also used a relativistic basis set on selenium. The transition from a double-ζ quality (6-31+G(d)) to a triple-ζ quality (6-311+G(2d)) basis set with a second polarization function at the geometry optimization stage leads to noticeable improvements (*R*^2^ = 0.969, Figure 3, Table S1), although not radical. This is especially evident on the models in the low-field area; for them there is approximately the same scatter (Figure S2a).

In principle, for selenium, one cannot exclude the influence of relativistic effects on its CS [34,35,36,37]. As a first approximation, we tried to identify these effects within the framework of a scalar relativistic approach using an all-electron relativistic triple-ζ doubly polarized basis set (NMR-DKH) on selenium [41,42], while on other elements the above-mentioned basis sets were used. The correlation of the data obtained in this way with the experiment is almost the same (*R*^2^ = 0.969, Figure 3, Table S1); this is especially evident for resonances in the low-field region, for which the characteristic scatter in the correlation dependence remains (Figure S2b). It is noteworthy that at the ends of the spectral range there is an overestimation of CS values in absolute value, i.e., as if some systematic errors appear. In general, at this level of theory, no relativistic effects were identified for these systems. Moreover, within this approximation for the standard (Se(CH_3_)_2_), a shift to low fields (by 95 ppm) is expected compared to the non-relativistic approach (Table S1).

The next step the basis sets was augmented at the shielding calculation (6-311G(3df,2p)) using the PBE0/6-311+G(2d) geometry. However, this gave only a slightly noticeable effect (*R*^2^ = 0.969, Figure 3, Table S1). Further augmenting of the basis set at the geometry optimization stage (PBE0/6-311G(3df,2p)//PBE0/6-311+G(3df,2p)) did not lead to the positive result (*R*^2^ = 0.968, Figure 3, Table S1). We also used the NMR-DKH basis on selenium in combination with this fairly flexible basis set on other elements (PBE0/6-311G(3df,2p);Se(NMR-DKH))//PBE0/6-311+G(3df,2p) Se(NMR-DKH))). In this case, a slight increase in the correlation coefficient was observed (*R*^2^ = 0.975, Figure 3, Table S1), although the variation for resonances in the low-field region remained approximately the same (Figure S2c). An overestimation of ^77^Se CSs in absolute terms was also observed, especially in the low-field region.

Thus, the effect of the basis set at the geometry optimization stage on chemical shifts is observed only when moving from a double-ζ quality to a triple-ζ quality basis set and then there are practically no changes. Interestingly, this trend correlates quite well with the influence of the basis set on the geometric parameters. Specifically, significant changes in the key geometrical parameters involving the Se atom (Se-C bond distance, N-C-Se and N-C-N angles) are also observed during the transition from a double-ζ quality to a triple-ζ quality basis set and further changes are minimal (Table S3), i.e., a sort of basis set limit for geometry is reached. Moreover, replacing the basis set on selenium with a relativistic one (NMR-DKH) does not practically affect the geometry (Table S3).

To summarize, we can say that simply increasing the flexibility of the basis sets both at the stage of geometry optimization and the stage of shielding calculation does not solve the problem of the scatter in correlation dependence, and the problem lies in something else.

#### 2.1.4. Variation of Functional Parameters

Until now, we have used the popular PBE0 functional, which has shown its effectiveness in a wide range of NMR shift calculations. To see how the functional parameters affect the ^77^Se shift, we varied the proportion of the exact exchange admixture at the shielding calculation stage, since it can have a strong influence especially for systems with a large paramagnetic term [30,43,44]. Thus, two cases were analyzed: when this contribution is minimal, i.e., with pure GGA PBE functional, and when the exact exchange contribution is increased to 50% (PBE50). The use of pure PBE functional leads to a noticeable deterioration in the correlation between calculation and experiment (*R*^2^ = 0.949 vs. 0.969 (PBE0), Figure 3, Table S1). In the case of the PBE50 functional, changes are less (*R*^2^ = 0.967 vs. 0.969 (PBE0)). At the same time, the characteristic scatter of data, especially for the low-field region, remains the same (Figure S3). Thus, such modifications of the functional do not lead to the desired correction for problematic cases; that is, apparently, a change in the parameters of the functional at shielding calculation cannot be used to solve the above problem.

We also checked how the type of functional (pure vs. hybrid) at the optimization stage may affect agreement between calculation and experiment. Thus, we carried out calculations using the pure PBE functional for optimization, while at the shielding calculation, a hybrid PBE0 functional was tested. In this case, notable worsening of correlation was also observed (*R*^2^ = 0.958, Figure 3, Table S1) although it was less than when this functional was used for shielding calculation. Finally, the worst result was observed when this pure functional was used at both stages of calculation (*R*^2^ = 0.948, Figure 3, Table S1). Thus, pure PBE functional has limitations, especially at the stage of shielding calculation.

#### 2.1.5. Full-Relativistic Correction to the ^77^Se NMR Shift

The reason for such a large dispersion, especially for low-field resonances, may be due to the presence of relativistic effects that are not captured within the scalar (1c) approximation. Therefore, we carried out calculations on representative adducts for which characteristic signals are observed in high- and low-field regions at a fully relativistic matrix Dirac–Kohn–Sham (mDKS) level [45].

For these calculations, two sets of geometries were used, obtained by optimization at a completely non-relativistic level (PBE0/6-311+G(2d)) and by replacing the basis set on selenium with an NMR-DKH basis set and an augmenting basis sets on elements (PBE0/6-311+G(3df,2p);Se(NMR-DKH)). In both cases Dyall’s valence double-ζ basis set [46] was used for the Se center and a locally dense basis set schemes was employed for other elements [22]. Specifically, we used large triple-ζ quality basis sets (ucc-pVTZ) on vicinal atoms to selenium (carbene carbon and two nitrogens), which are important in the calculation, and smaller double-ζ quality basis sets (ucc-pVDZ) on the other atoms (denoted as “TZ_DZ”).

In the first case, the fully relativistic data were downfield shifted by ca. 25–30 ppm in respect to non-relativistic results (PBE0/6-311G(2d,2p)//PBE0/6-311+G(2d), Table S4). However, it is important that the overall picture remains approximately the same as before at the non-relativistic level. This is especially clearly seen in the example of problematic low-field resonances (Figure S4a). Thus, in the ^77^Se NMR chemical shifts scale, the total relativistic corrections are deshielding (in contrast to shielding relativistic corrections to selenium shielding constants), which is because Se(CH_3_)_2_ (a standard) has large relativistic corrections (Δσ_rel_ = 278 ppm, according to our calculations, Table S4) that are in reasonable agreement with the literature data (Δσ_rel_ = 262 ppm [22]).

Just in case, we also tried to augment the basis set for the Se atom up to Dyall’s valence triple-ζ one (denoted as “DTZ_TZ_DZ”). In this case, the overall picture remained the same, although the apparent relativistic effects (low-field shifts) were somewhat smaller due to the reduction of relativistic corrections for the standard (Δσ_rel_ = 246 ppm, Table S4).

At the same time, for the NMR-DKH geometry, the difference between the methods was noticeably higher. Specifically, in this case, relativistic correction for a standard (Se(CH_3_)_2_) was larger (Δσ_rel_ = 376 ppm, Table S4). Approximately the same corrections were predicted for low-field resonances, while in high fields the corrections were expected even larger. However, the general pattern of scatter, especially for low-field signals, remained unchanged (Figure S4b). Thus, the reason for the scatter in the correlation for low-field resonances is not due to different relativistic corrections for these compounds.

#### 2.1.6. Some Final Remarks on ^77^Se NMR Shift Calculations of NHC–Selenoureas

Varying the computational approximations within the framework of the isolated molecule model leads to approximately the same scatter in the correlation between the calculated and experimental data. This variation does not depend on the basic set, functionality and level of theory used.

Taking into account the tendency of Se to NCHB, it can be assumed that the structural features due to intermolecular HB with solvent also play a role. For example, calculations for some problematic carbene Se adducts with the inclusion of chloroform explicitly show that such structures indeed correspond to a minimum energy (gain ca. 4 kcal/mol), and for them a resonance shift to high field by ca. 20 ppm is expected (Table S5). However, the exact population of such complexes cannot be predicted well. Therefore, such a variation in correlation of calculated vs. experimental data can be attributed to a random error that occurs due to the impact of the supramolecular structure, and which cannot yet be taken into account or foreseen.

**Figure 4:**
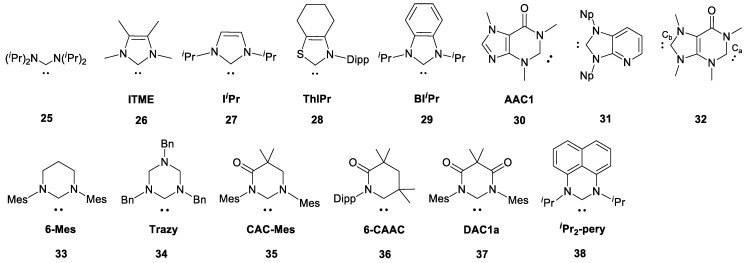
Additional NHCs set (**25**–**38**) for ^77^Se shift calculations.

**Figure 5:**
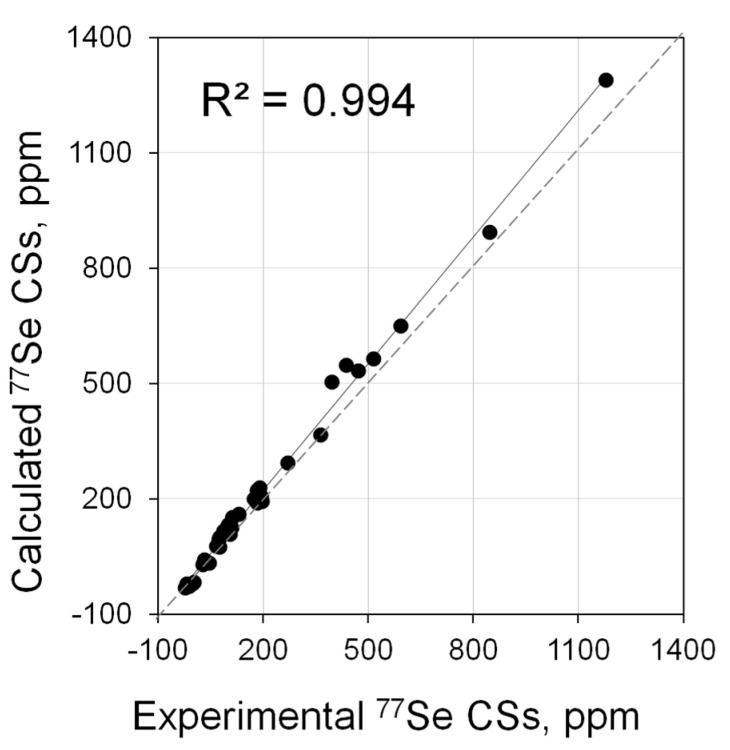
Correlation of calculated (PBE0/6-311G(2d,2p)//PBE0/6-311+G(2d)) vs. experimental ^77^Se NMR shifts for the extended set of carbene Se adducts (**1**·Se–**38**·Se).

The next three protocols (PBE0/6-311G(2d,2p)//PBE0/6-31+G(d), PBE0/6-311G(2d,2p)//PBE0/6-311+G(2d) and PBE0/{6-311G(3df,2p);Se(NMR-DKH)}//PBE0/{6-311+G(3df,2p);Se(NMR-DKH)}) were tested on a more extended test set of NHC-derived selenoureas (**25**·Se–**38**·Se, Figure 4) for which ^77^Se shifts were observed in significantly lower-field regions (up to 1200 ppm, some data in acetone, Table S6). All three approximations reproduced the experimental data quite well in such a wide CS range (Figure 5). The maximum correlation coefficient (*R*^2^ = 0.994, Table S6) was observed when the PBE0/6-311G(2d,2p)//PBE0/6-311+G(2d) combination was used.

Thus, ^77^Se NMR shifts of carbene Se adducts can be calculated quite well within the framework of non-relativistic approximations. The PBE0/6-311G(2d,2p)//PBE0/6-311+G(2d) approximation is optimal.

However, there are certain limitations associated with the tendency of selenium to form NCHBs, leading to the stabilization of additional forms, the ^77^Se NMR shifts of which are very different. The population of such forms is difficult to estimate accurately, which makes it difficult to correctly assess the contribution of each form to the exchange average NMR spectrum. This factor, apparently, is the main one limiting the accuracy of the prediction.

A strong anomalous solvent effect was also found within the PCM model, especially at the shielding calculation stage. Therefore, from a practical point of view, it seems more reliable to carry out calculations for vacuum.

Using the PBE functional as an example, it was shown that pure GGA functionals have limitations and are less reliable, especially at the shielding calculation stage.

For carbene Se adducts, relativistic corrections in shielding are expected to be quite high, but in the ^77^Se NMR chemical shifts scale, the total relativistic corrections are deshielding due to dimethyl selenide (a standard) possesses large relativistic correction. These corrections in ^77^Se NMR shifts are small (25–30 ppm) and approximately the same for all considered carbene Se adducts.

### 2.2. ^31^P NMR Shifts of NHC–Phosphinidene Adducts

One of the most promising approaches for evaluating the electronic properties of NHC is the method based on the ^31^P NMR shift of the corresponding easily synthesized phenylphosphinidene–carbene adducts. This is due both to the relatively high absolute sensitivity of the ^31^P NMR signal and to the presence of attractive schemes for interpreting changes in spectra within the framework of simple, intuitive resonance structures [11,20,21,47]. In this case, the range of changes in ^31^P CSs of NHC–phosphinidene adducts is smaller than in the case of ^77^Se. However, perhaps in this case the NMR parameters would not be modulated by additional effects and would only be a function of the electronic distribution of a certain structural form. Therefore, we tried to find out the key factors (structural, conformational, parameters of the computational approximation) that determine the actual agreement of the calculation and experiment.

**Figure 6:**
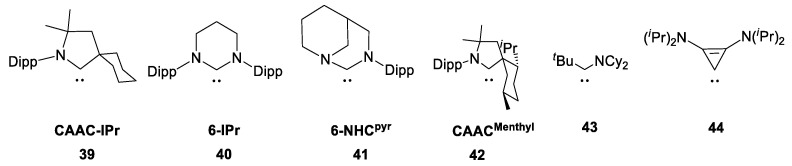
Structures of NHCs **39**–**44**.

In the first stage as the main training set (**3**, **5**, **11**, **13**, **26**–**29**, **35**, **39**–**40**, Figure 6), we considered the same models used for ^31^P CS calculations in ref. [20]. Shielding calculations at the BP86/TZ2P//BP86/TZ2P level showed that the theory is reproduced in the experiment quite well (*R*^2^ = 0.987) [20]. However, in our opinion, these results can be improved. GGA functionals have limitations for calculating NMR shifts for systems in which the paramagnetic contribution is significant [30,43,44]. In NHC–phosphinidene adducts, such a contribution should be important. Therefore, to test this assumption, we performed ^31^P NMR shift calculations using the hybrid functional (PBE0). Specifically, the PBE0/6–311G(2d,2p)//PBE0/6–31+G(d)) combination was used, which proved itself well earlier for the calculation of ^31^P CSs in organophosphorus compounds [28].

**Figure 7:**
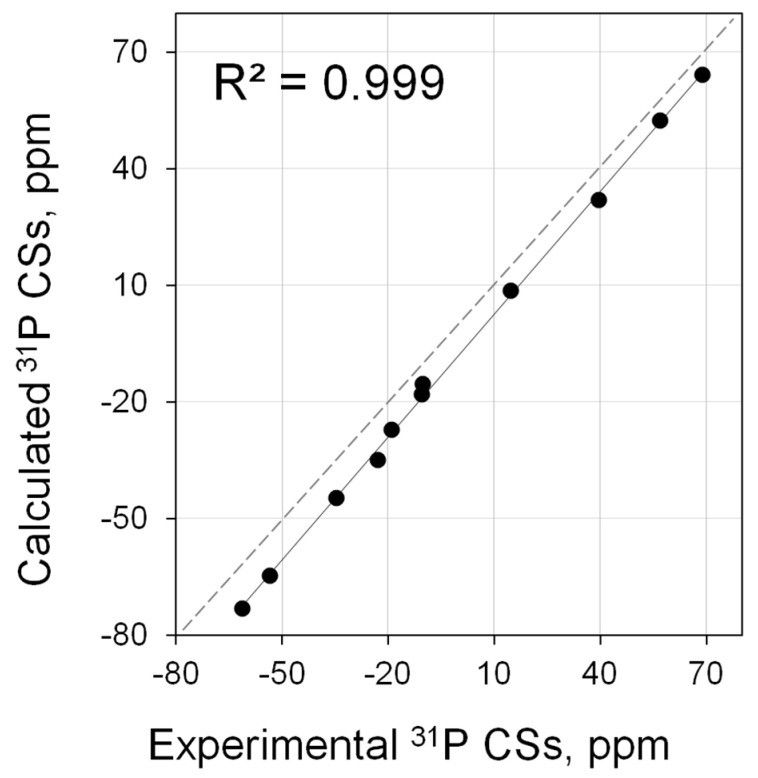
Correlation of calculated (PBE0/6-311G(2d,2p)//PBE0/6-31+G(d)) vs. experimental ^31^P NMR shifts for main set of carbene PPh adducts (**3**·PPh, **5**·PPh, **11**·PPh, **13**·PPh, **26**·PPh–**29**·PPh, **35**·PPh, **39**·PPh–**40**·PPh).

The calculation actually reproduces ^31^P CSs of NHC–phosphinidene adducts significantly better (*R*^2^ = 0.999, Figure 7, Table S7). This leads to a noticeable increase in accuracy in the characterization of NHC in terms of ^31^P CSs.

The inclusion of additional models (**25**, **37**, **41**–**44**, some data in polar solvents) in the analysis, including those for which ^31^P resonances were observed in lower fields (up to 126 ppm) leads to a good correlation of the calculation with the experiment, although slightly worse than for the original set (*R*^2^ = 0.992, Figure S5, Table S8).

To improve the agreement between theory and experiment, additional calculations were carried out by augmenting the basis set at the geometry optimization (PBE0/6-311G(2d,2p)//PBE0/6-311+G(2d)). For this extended set, some improvement was obtained (*R*^2^ = 0.993, Table S8). Augmenting the basis set at the shielding calculation stage (PBE0/6-311G(3df,2p)//PBE0/6-311+G(2d)) practically did not change the correlation (*R*^2^ = 0.993, Table S8). Therefore, from a practical point of view, the PBE0/6-311G(2d,2p)//PBE0/6-31+G(d) combination can be recommended.

Thus, this is a highly reproducible descriptor that is minimally contaminated by additional effects and primarily reflects the electronic structure of the NHC–phosphinidene adducts themselves. Some systematic errors inherent to DFT methods can be easily corrected by linear scaling procedure (vide infra).

### 2.3. ^13^C NMR Shifts of NHC–Phosphinidene Adducts

Although the ^13^C NMR chemical shift of the former carbene carbon was not considered as NMR metric of NHCs in the carbene·PPh adducts, it can also be very promising to this end since it varies in quite a wide range (from 138 up to 220 ppm, Table S8) depending on the parent NHC. In principle, this carbon could be more interesting to correlate with reactivity, since it is part of the original NHC and may more closely reflect the features of the electronic structure of the NHC. In this regard, it is interesting to see whether the theory reproduces such changes in ^13^C CS and what other factors may contribute to the observed ^13^C shift.

**Figure 8:**
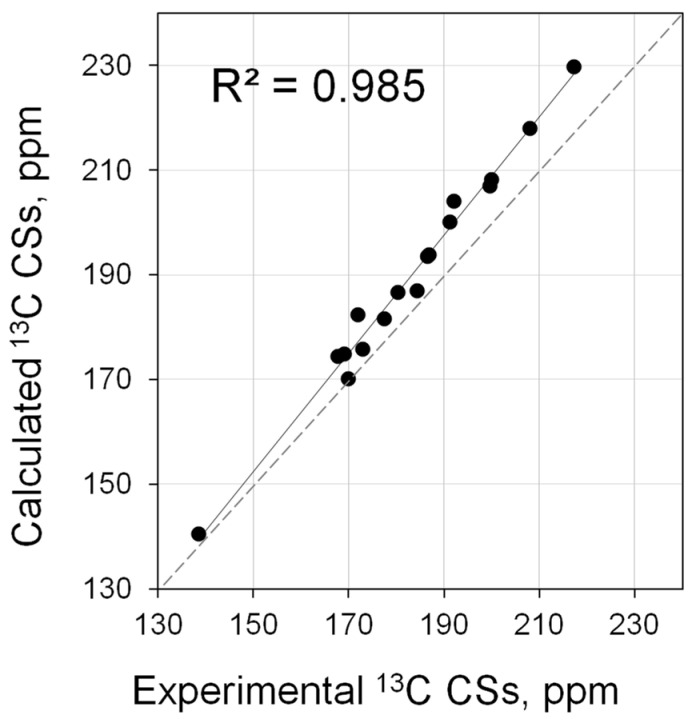
Correlation of calculated (PBE0/6-311G(2d,2p)//PBE0/6-31+G(d)) vs. experimental ^13^C NMR shifts for carbene PPh adducts (**3**·PPh, **5**·PPh, **11**·PPh, **13**·PPh, **25**·PPh–**29**·PPh, **35**·PPh, **37**·PPh, **39**·PPh–**44**·PPh).

Calculations at the PBE0/6-311G(2d,2p)//PBE0/6-31+G(d) level showed that this NMR metric can be calculated quite well (Table S8). Theoretical values correlate well with the experiment (Figure 8, *R*^2^ = 0.985, Table S8). Interestingly, the exceptional high-field shift of the carbene carbon for **44**·PPh was also reproduced.

The use of more flexible basis sets at the geometry optimization (PBE0/6-311G(2d,2p)//PBE0/6-311+G(2d)) or at the shielding calculations (PBE0/6-311G(3df,2p)//PBE0/6-311+G(2d)) leads only to negligible improvements (*R*^2^ = 0.986, Table S8). Thus, in this case, a rather modest combination (PBE0/6-311G(2d,2p)//PBE0/6-31+G(d)) can be recommended, since further enhancement does not lead to improvement. These calculations also suffer from systematic errors inherent in the DFT method in areas far from the reference. However, they are easily corrected using the linear scaling procedure (see below).

### 2.4. ^13^C Shifts of NHCs

Another NMR parameter that has practically not been used in the literature as a metric for comparing electronic properties of NHC is the ^13^C NMR shift of the carbene carbon. Perhaps this was due to the fact that, on the one hand, the signal of this carbon in ^13^C NMR spectra is usually low-intensity. On the other hand, the presence of a number of signals from other NHC carbon atoms in this region may lead to difficulties in reliably assigning of signals. However, modern ^1^H-^13^C HMBC experiments [48] make it possible to overcome both of these problems and open access to another NMR parameter that can be used to analyze the electronic properties of NHC.

**Figure 9:**
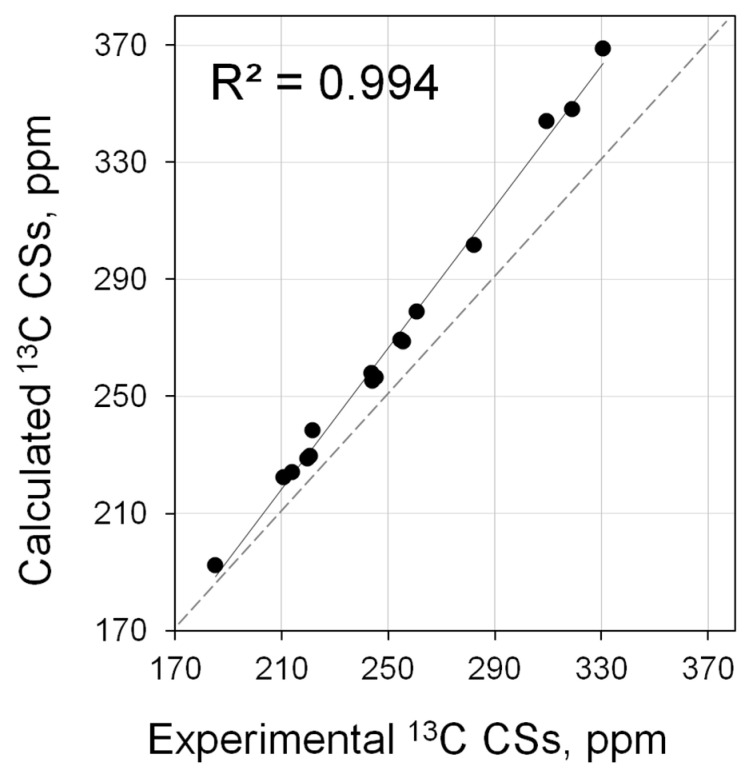
Correlation of calculated (PBE0/6-311G(2d,2p)//PBE0/6-31+G(d)) vs. experimental ^13^C NMR shifts for carbenes (**3**, **5**, **11**, **13**, **25**–**29**, **35**, **39**–**44**).

Calculations within the already well-established approximation (PBE0/6-311G(2d,2p)//PBE0/6-31+G(d)) showed that the theory reproduces experiment very well (Figure 9). There is an almost perfect correlation between calculation and experiment (*R*^2^ = 0.994, Table S9). A slight underestimation of shielding is to be expected for ^13^C shifts in this region due to systematic errors inherent in the DFT method. However, the use of the linear scaling procedure (vide infra) can significantly improve the quantitative agreement between calculation and experiment. Thus, ^13^C of NHC carbene carbon is a perfectly reproducible descriptor. Moreover, in this case, two advantages are expected: on the one hand, we are dealing with practically undisturbed NHC, the NMR parameters of which may be directly related to the electronic properties of NHC. On the other hand, the carbene carbon is actually at the epicenter of the reaction center and its CS may be most directly related to the electronic structure of this center. In principle, it is clear from the available data that the range of changes in the CS of this carbon is quite large (180–330 ppm, Table S9), i.e., this is a fairly sensitive indicator.

### 2.5. ^13^C Shifts of NHC–Azolium Salts

One more NMR metric that potentially could be used to gauge the electronic properties of the respective NHCs is ^13^C carbene shift of an azolium salt. Azolium salts are the most common NHC precursors and can be easy accessed. Only the corresponding heteronuclear interaction constant ^1^*J*(C-H) for the C-2 atom in a cationic carbene was used to characterize the electronic properties of carbenes [11,18]. However, so far, the methods for calculating spin–spin couplings have not advanced as much as the approaches for calculating CSs, so this parameter is difficult to predict with good accuracy. At the same time, ^13^C shifts can be estimated quite well, unless additional effects indirectly affect this value.

**Figure 10:**
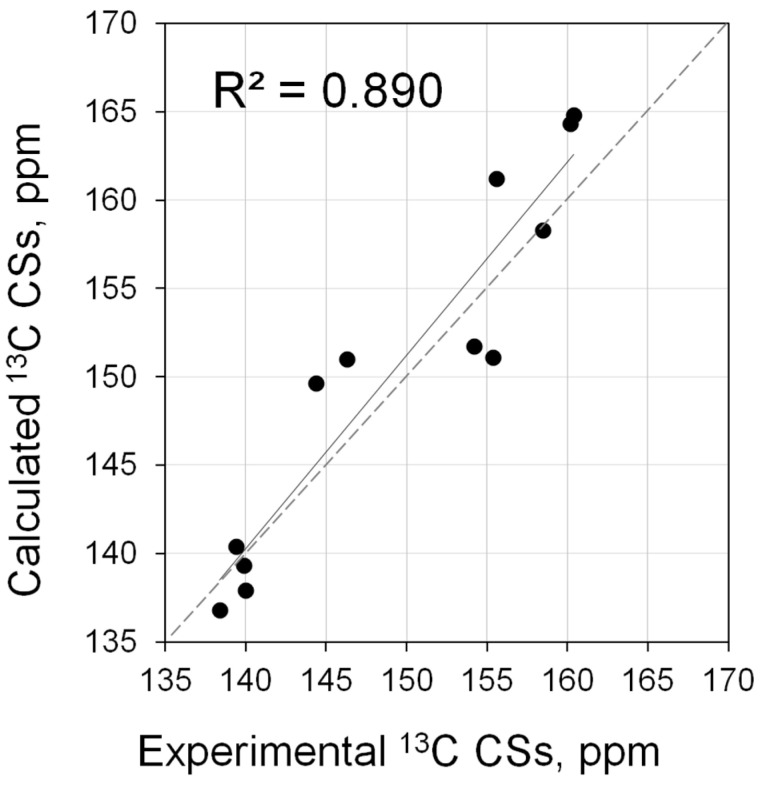
Correlation of calculated (PBE0/6-311G(2d,2p)//PBE0/6-31+G(d)) vs. experimental ^13^C NMR shifts for carbene (**3**·H^+^, **5**·H^+^, **11**·H^+^, **17**·H^+^, **28**·H^+^–**30**·H^+^, **32**·H^+^–**35**·H^+^).

In general, it can be noted that the changes in ^13^C CSs of the C-2 atom in the cationic carbene are noticeably less (<20 ppm, Table S10) than for the parent carbene itself or even phosphinidene adducts. The calculations showed that, in general, the theory reproduces the trend (Figure 10), but the correlation is much worse (*R*^2^ = 0.890, Table S10).

This scatter can be easily explained by the presence of fast exchange (on the NMR time scale) in solution with both the deprotonated form and the form associated with the counterion. In solution, the population of each of these forms is difficult to assess accurately; therefore, given the fact that their spectral parameters are very different, the parameters in the averaged NMR spectrum can vary noticeably. Moreover, the concentration of the substance, the type of the solvent and the temperature can also influence these equilibrium processes, introducing additional error. Thus, this NMR characteristic is, in principle, poorly predictable. In this regard, estimates of the coupling constant ^1^*J*(C–H) for the C-2 atom will also suffer from these factors.

### 2.6. Huynh’s Electronic Parameter (HEP)

Huynh introduced a methodology based on ^13^C NMR spectroscopy of NHC complexes [PdBr_2_(*^i^*Pr_2_-bimy)*L*] (*^i^*Pr_2_-bimy = 1,3-diisopropylbenzimidazolin-2-ylidene) used to measure the donor ability of the *trans*-ligand *L* [11,15,16]. In essence, the influence of a *trans* standing ligand *L* on the chemical shift of ^13^C_carbene_ NMR signal of the *^i^*Pr_2_-bimy reporter ligand (i.e., HEP value) is measured.

It was noticed that a stronger donor ligand induces a downfield shift, while a weaker donor results in an upfield shift of the ^13^C carbene NMR signal of the *^i^*Pr_2_-bimy spectator ligand. The authors proposed the model, according to which a stronger *trans* donor *L* weakens the Pd–*^i^*Pr_2_-bimy bond more so than a weaker one, leading to an enhanced “free *^i^*Pr_2_-bimy” character, which leads to a downfield shift.

However, it was shown recently that the mechanism of influence of the *trans*-ligand on the ^13^C carbene NMR shift is somewhat different [33]. Specifically, the σ-donating ability of the ligand *L* affects the electronic structure of the metal-*trans*-NHC carbon bond, which in turn modulates the relativistic spin–orbit (SO) effects of palladium on the NMR shift of that carbon.

In general, changes in HEP values are not very large (25–35 ppm, Table S11). In principle, this is expected since HEP values are modulated by the relativistic SO effect of the metal, which depends on the covalency of the metal–NHC bonds, which are not much different on the two sides of the metal.

**Figure 11:**
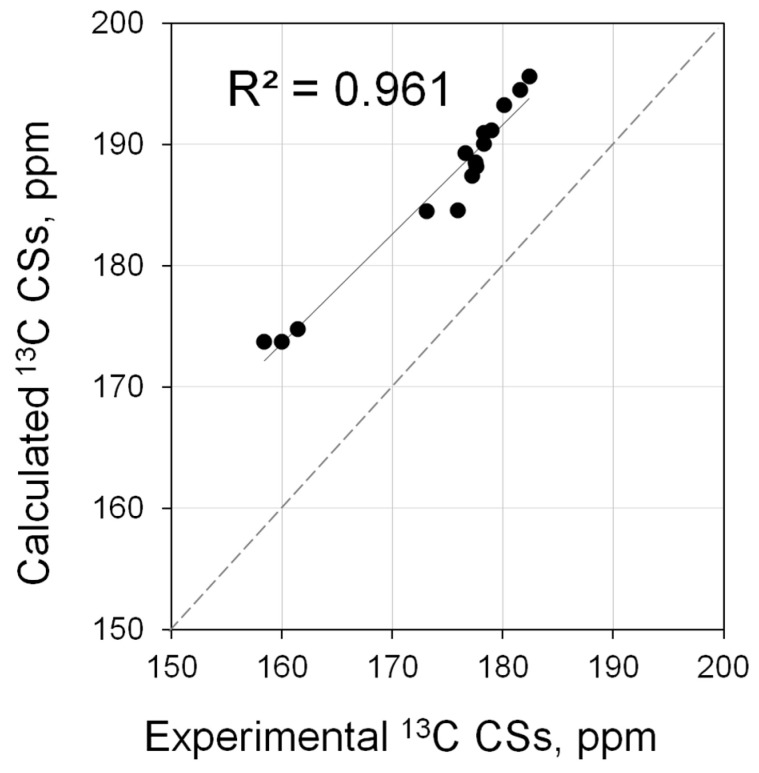
Correlation of calculated (at mDKS level) vs. experimental ^13^C NMR shifts for the carbene atoms in [PdBr_2_(*^i^*Pr_2_-bimy)*L*] complexes (*L* = **3**, **5**, **11**, **45**–**56**).

The possible effects were assessed for a number of model structures for which fully relativistic calculations were affordable (**3**, **5**, **11**, **45**–**56**, Figure S6). It was found that at a fully relativistic matrix Dirac–Kohn–Sham (mDKS) level, there is some correlation between theory and experiment (Figure 11) although it is not perfect (*R*^2^ = 0.961, Table S11). Unfortunately, in this case, it is not yet possible to improve the correlation of calculations with experiment by varying the parameters, since there are significant limitations on computational resources for such calculations.

### 2.7. Minimization of Systematical Errors—Empirical Linear Scaling Procedure

Thus far, the correlation coefficient has been considered as a criterion for the quality of calculation, i.e., the better the linear relationship between theory and experiment, the better the correlation can be predicted. However, the *R*^2^ value characterizes only the point scattering relative to the regression line but not the real accuracy of the chemical shift values. If we compare the absolute values, it can be seen that in most cases there are systematic errors inherent in DFT methods, which increase with distance from the reference. Moreover, in some cases, such as ^77^Se NMR, the calculated shielding of the generally accepted standard (Se(CH_3_)_2_) is strongly dependent on the level of theory, which leads to an additional systematic error in the calculated values.

One way to minimize such errors is to use secondary standards; compounds of a similar nature and structure (for example, benzene for ^13^C NMR shifts in the aromatic region and some NHC–selenium adducts for ^77^Se NMR shifts). However, the most general approach to error reduction is empirical linear scaling, specifically, the application of corrections (scaling factors) derived from linear regression procedures [22,49,50]. Thus, CSs can be recalculated according to Equation (1)

δ_scaled_ = (δ − Intercept)/Slope, (1)

where δ is the calculated CS for a particular nucleus and Intercept and Slope are scaling factors. The scaling factors were derived from linear regression procedures on the corresponding training sets (Table 1). The intercept fixes the error for a reference compound, while the slope helps to correct the systematic errors. Thus, the application of this procedure allows for the evaluation of NMR shifts that can be compared with experimental ones quantitatively and to determine Root Mean Square Errors (*RMSEs*) that characterize real random error. In this case, it becomes possible to estimate the scatter due to a random error, which, in fact, determines the uncertainty in the NMR characteristic for each of the NMR-based methods. This is the accuracy with which any particular NMR metrics can be assessed. The *RMSE*s thus obtained are given in Table 1.

### 2.8. Practical Aspects

The overview of the presented data allows us to draw certain conclusions about the scopes and limitations of NMR-based metrics. Calculations of the ^77^Se shifts of NHC-derived selenoureas are characterized by an *RMSE* of ca. 20 ppm for the ^77^Se NMR chemical shift range reaching 1200 ppm, resulting in a very low percentage error of 1.6% (Table 1). That is, the calculations reproduce well and reliably the strong variation in the electronic characteristics of NHC adducts, which is expressed in such a strong change in ^77^Se NMR shifts. However, if we consider the data in the context of real problems of reliable NMR differentiation of closer structures, this approach has its limitations. Specifically, if, for example, we consider the results of statistical analysis for a narrower range of CSs (220 ppm) in a low polarity solvent (CHCl_3_), the relative error increases (5.3%, Table 1). Thus, it seems that more subtle differences cannot be predicted reliably in terms of ^77^Se NMR shifts. This limitation is due to the presence of additional modulation of ^77^Se NMR parameters due to structures with NCHBs, the contribution of which cannot be accurately assessed.

In this regard, methods based on the analysis of NMR parameters of NHC–phosphinidene adducts look more promising. Specifically, calculations of ^31^P NMR shifts for these derivatives lead to an *RMSE* of 4.3 ppm for the range of chemical shifts 190 ppm, which gives a fairly low percentage error of 2.3% (Table 1). However, if we limit ourselves to a narrower range with accurate experimental data in a low polarity solvent (CHCl_3_), then we get a record low *RMSE* of 1.5 ppm and the lowest percentage error of 1.1% (Table 1). In other words, this method can reliably predict small differences in the electronic properties of carbene•PPh adducts. The ^13^C NMR shifts for these adducts are also quite reproducible (2.8%, Table 1) and look promising for the analysis of such carbenes.

The method based on the ^13^C NMR shift of the carbene carbon also worked quite well. It is estimated that this results in a low percentage error of 2.1% (Table 1). Taking into account the fact that for this method there is no need for additional preparation reactions, that we are dealing with an unperturbed NHC and that this carbon is actually the reaction center, this approach may turn out to be the most promising.

At the same time, methods based on the analysis of HEP, especially ^13^C shifts of carbene azolium salts, are noticeably inferior in accuracy to the approaches discussed above (Table 1). HEP is characterized by a fairly high percentage error of 6.8%. For carbene azolium salts, this parameter is even worse (13.5%).

## 3. Materials and Methods

### Calculations

The quantum chemical calculations were carried out within the framework of Kohn–Sham density functional theory [51], with the Gaussian 16 [52] (Revision A.03) software packages using PBE [53], PBE0 [54] and PBE50 functionals and a Pople’s basis sets [55,56,57,58,59,60,61,62]. For the Se atom, the triple -ζ doubly polarized NMR-DKH basis with all coefficients obtained from a Douglas–Kroll–Hess (DKH) second-order scalar relativistic calculation was used [41]. The energy of intermolecular interactions of NHC-derived selenoureas with CHCl_3_ was calculated as the sum of energies of components minus the energy (BSSE corrected) of the supramolecular associate. BSSE corrections were performed using the counterweight method [63]. To take into account the medium effects, calculations were also carried out in the framework of the polarizable continuum model [40] (denoted as “PCM”). NMR CSs were calculated by the GIAO method [64]. All ^77^Se, ^31^P and ^13^C data were referenced to dimethyl selenide (Se(CH_3_)_2_), phosphoric acid (H_3_PO_4_) and tetramethylsilane (TMS, Si(CH_3_)_4_) respectively, which were calculated under the same conditions. The NMR-DKH basis sets were downloaded from the EMSL basis set library for the Gaussian package [42].

Fully relativistic DFT ^77^Se CS calculations were carried out at the matrix Dirac–Kohn–Sham (mDKS) [45] level with the ReSpect-MAG code [65]. The four-component mDKS calculations were done with the PBE0 functional. The uncontracted Dyall [46] valence double- and triple -ζ basis set for the Se atom was used. For some elements, a locally dense basis set schemes was employed [22]: large triple-ζ quality basis sets (ucc-pVTZ) on vicinal atoms to selenium (carbene carbon and two nitrogens) and smaller double-ζ quality basis sets (ucc-pVDZ) on the other atoms. For these calculations, two sets of geometries were used, obtained by optimization at a completely non-relativistic level (PBE0/6-311+G(2d)) and by replacing the basis set on selenium with the DKH recontracted all-electron NMR-DKH basis set and augmenting basis sets on elements (PBE0/6-311+G(3df,2p);Se(NMR-DKH)).

For palladium complexes, ^13^C shift calculations (HEP) were carried out at a fully relativistic mDKS level of theory [45]. The uncontracted Dyall [46] valence double-ζ basis set was used for the Pd center. For ligand atoms, triple-ζ quality basis sets (ucc-pVTZ) on spectator atoms and vicinal atoms to Pd center and double-ζ quality basis sets (ucc-pVDZ) on the remaining atoms were used. For these calculations, the PBE0/{6-31+G(d); Pd(SDD)} geometry was used.

## 4. Conclusions

The scopes and limitations of theoretical methods for estimating various NMR metrics of NHCs are considered on a wide range of model NHCs and their derivatives, using a number of computational approximations, including non-relativistic and relativistic levels of theory, with variations of basis sets and functionals, with and without taking into account the solvent effects.

The most reliable, from the point of view of predictability, are ^31^P/^13^C NMR shifts of NHC–phosphinidene adducts and ^13^C CSs of carbenes themselves.

The method based on the analysis of ^77^Se CS NHC-derived selenoureas has some limitations since the observed NMR parameters, in addition to depending on the electronic structure, can also be modulated by exchange effects due to forms with NCHBs.

As for HEP, since the delicate balance of electron distribution between Pd and two carbon centers can nonlinearly affect relativistic SO effects, the accuracy of the estimation of this metric may still be limited.

^13^C CSs of NHC–azolium salts do not seem to be reliable, since the observed values are strongly influenced by the effects, due to the exchange between different forms with counterions, which are difficult to estimate correctly.

## Figures and Tables

**Table 1:** Linear regression parameters for correlation of calculated a vs. experimental ^77^Se, ^31^P and ^13^C NMR shifts for carbene·Se adducts, carbene·PPh adducts, carbenes, carbene·azolium salts and *trans*-[PdBr_2_(*^i^*Pr_2_-bimy)*L*] adducts.

	Nuclei	Range, ppm	Level ^b^	Slope	Intercept	*R* ^2^	*RMSE*, ppm	*nRMSE* ^c^, %
Carbene·Se adducts	^77^Se	219.0 ^a^	A	1.20	−27.16	0.951	14.7	6.7
			B	1.15	−0.55	0.969	11.5	5.3
			C	1.29	−6.59	0.975	10.3	4.7
		1202.1	A	1.12	−19.53	0.991	23.8	2.0
			B	1.10	4.59	0.994	19.3	1.6
			C	1.15	3.56	0.993	21.0	1.7
Carbene·PPh adducts	^31^P	130.1 ^a^	A	1.05	−7.99	0.999	1.5	1.1
			B	1.05	−1.49	0.999	1.6	1.2
			D	1.07	−2.44	0.998	1.9	1.5
		187.5	A	1.11	−7.96	0.992	4.8	2.6
			B	1.09	−1.61	0.993	4.3	2.3
			D	1.10	−2.79	0.993	4.3	2.3
	^13^C	78.7	A	1.13	−17.09	0.985	2.2	2.8
			B	1.11	−14.87	0.985	2.2	2.8
			D	1.11	−12.73	0.986	2.1	2.7
Carbene	^13^C	145.4	A	1.21	−34.84	0.994	3.0	2.1
Carbene·H^+^ azolium salts	^13^C	22.0	A	1.09	−12.95	0.890	3.0	13.5
*trans*-[PdBr_2_(*^i^*Pr_2_-bimy)*L*]	^13^C	24.0	E	0.90	29.49	0.961	1.6	6.8

^a^ main set; ^b^ A—PBE0/6-311G(2d,2p)//PBE0/6-31+G(d) level, B—PBE0/6-311G(2d,2p)//PBE0/6-311+G(2d) level, C—PBE0/{6-311G(3df,2p); Se(NMR-DKH)}//PBE0/{6-311+G(3df,2p); Se(NMR-DKH)} level, D—PBE0/6-311G(3df,2p)//PBE0/6-311+G(2d) level, E—4c-mDKS: PBE0/{ucc-pvtz+ucc-pvdz; Pd(dyall-vdz)}//KS: PBE0/{6-31+G(d); Pd(SDD)} level; ^c^ *nRMSE* = *RMSE*/Range × 100%.

## Data Availability

All data are contained within the article and Supplementary Materials.

## Supplementary Materials

The following supporting information can be downloaded at: https://www.mdpi.com/article/10.3390/molecules28237729/s1, Figure S1: Different forms of I^i^Pr^Me^·Se (**6**·Se) with corresponding distances (Å) between *^i^*Pr hydrogen’s and Se; Figure S2: Correlation of calculated vs. experimental ^77^Se NMR shifts for the main set of carbene Se adducts (**1**·Se–**24**·Se) (a—PBE0/6-311G(2d,2p)//PBE0/6-311+G(2d) level, b—PBE0/{6-311G(2d,2p); Se(NMR-DKH)}//PBE0/{6-311+G(2d); Se(NMR-DKH)}, c—PBE0/{6-311G(3df,2p); Se(NMR-DKH)}//PBE0/{6-311+G(3df,2p); Se(NMR-DKH)} level); Figure S3: Correlation of calculated vs. experimental ^77^Se NMR shifts for the main set of carbene Se adducts (**1**·Se–**24**·Se) (a—PBE/6-311G(2d,2p)//PBE0/6-311+G(2d) level, b—PBE50/6-311G(2d,2p)//PBE0/6-311+G(2d) level); Figure S4: Correlation of calculated vs. experimental ^77^Se NMR shifts for the main set of carbene Se adducts (a—**1**·Se–**2**·Se, **4**·Se–**7**·Se, **13**·Se–**14**·Se, **17**·Se–**20**·Se, TZ_DZ//PBE0/6-311+G(2d) level, b—**1**·Se–**2**·Se, **6**·Se, **13**·Se–**14**·Se, **17**·Se–**19**·Se, TZ_DZ//PBE0/{6-311+G(3df,2p); Se(NMR-DKH)} level); Figure S5: Correlation of calculated (PBE0/6-311G(2d,2p)//PBE0/6-31+G(d) level) vs. experimental ^31^P NMR shifts for extended set of carbene PPh adducts (**3**·PPh, **5**·PPh, **11**·PPh, **13**·PPh, **25**·PPh–**29**·PPh, **35**·PPh, **37**·PPh, **39**·PPh–**44**·PPh); Figure S6: Structures of [PdBr_2_(*^i^*Pr_2_-bimy)*L*] complexes. *L* = **3**, **5**, **11**, **45**–**56**; Table S1: Experimental (in CDCl_3_) and calculated ^77^Se NMR shifts (ppm) for main set of carbene·Se adducts (**1**·Se–**24**·Se); Table S2: ^77^Se NMR shifts (ppm) for ICy (**1** Se), IDD (**2** Se) and I^i^Pr^Me^ (**6** Se) Se-adducts. Experimental (in CDCl_3_) and calculated CSs for different forms with corresponding energies (in parenthesis); Table S3: Se-C bond lengths (L, Å) and some valence angles (°) for **1**·Se, **5**·Se, **7**·Se, **13**·Se, and **18**·Se calculated at DFT level of theory (PBE0) with different basis sets; Table S4: Experimental (in CDCl_3_) and calculated ^77^Se NMR shifts (ppm) at non-relativistic and fully relativistic mDKS levels of theory for selected carbene·Se adducts (**1**·Se–**2**·Se, **4**·Se–**7**·Se, **13**·Se–**14**·Se, **17**·Se–20·Se); Table S5: Experimental (in CDCl_3_) and calculated ^77^Se NMR shifts (ppm) for some carbene·Se adducts (**13**·Se–**14**·Se, **17**·Se) and their associates with chloroform; Table S6: Experimental (in CDCl_3_ unless otherwise stated) and calculated ^77^Se NMR shifts (ppm) for extended set of carbene·Se adducts (**1**·Se–**38**·Se); Table S7: Experimental (in C_6_D_6_ unless otherwise stated) and calculated ^31^P NMR shifts (ppm) for main set of carbene·PPh adducts (**3**·PPh, **5**·PPh, **11**·PPh, **13**·PPh, **26**·PPh–**29**·PPh, **35**·PPh, **39**·PPh–**40**·PPh); Table S8: Experimental (in C_6_D_6_ unless otherwise stated) and calculated ^31^P and ^13^C NMR shifts (ppm) for extended set of carbene·PPh adducts (**3**·PPh, **5**·PPh, **11**·PPh, **13**·PPh, **25**·PPh–**29**·PPh, **35**·PPh, **37**·PPh, **39**·PPh–**44**·PPh); Table S9: Experimental (in C_6_D_6_) and calculated ^13^C NMR shifts (ppm) for set of carbenes (**3**, **5**, **11**, **13**, **25**–**29**, **35**, **39**–**44**); Table S10: Experimental (in DMSO-*d*_6_ unless otherwise stated) and calculated ^13^C NMR shifts (ppm) for set of carbene·H^+^ azolium salts (**3**·H^+^, **5**·H^+^, **11**·H^+^, **17**·H^+^, **28**·H+–**30**·H^+^, **32**·H^+^–**35**·H^+^); Table S11: Experimental (in CDCl_3_) and calculated ^13^C NMR shifts (ppm) of *^i^*Pr_2_-bimy carbenoid resonances in *trans*-[PdBr_2_(*^i^*Pr_2_-bimy)*L*] complexes.
