# Supplementary material for: NMR “Finger Prints” of N-Heterocyclic Carbenes, DFT Analysis: Scopes and Limitations
Source: Molecules. 2023 Nov 23;28(23):7729. doi: 10.3390/molecules28237729 (PMC10707865; doi:10.3390/molecules28237729)
Supplement: Supplementary file 1 [file molecules-28-07729-s001.zip › molecules-2704789-supplementary.pdf]

Supplementary Information for:

NMR "finger prints" of N-heterocyclic carbenes. DFT analysis. Scopes and limitations.

*Svetlana A. Kondrashova, Shamil K. Latypov**

Arbuzov Institute of Organic and Physical Chemistry, FRC Kazan Scientific Center of RAS,
Kazan, Tatarstan 420088, Russian Federation

* *Correspondence: lsk@iopc.ru; Tel.: +7-(843)-2731892; Fax: +7-(843)-2732253*

Table of contents **pages**

Table S1. Experimental (in $CDCl_3$) and calculated ^{77}Se NMR shifts (ppm) for main set of carbene·Se adducts (**1**·Se-**24**·Se). 3

Figure S1. Different forms of **I^{i}Pr^{Me}**·Se (**6**·Se) with corresponding distances (Å) between ^{i}Pr hydrogen's and Se. 5

Table S2. ^{77}Se NMR shifts (ppm) for **ICy** (**1** Se), **IDD** (**2** Se) and **I^{i}Pr^{Me}** (**6** Se) Se-adducts. Experimental (in $CDCl_3$) and calculated^a CSs for different forms with corresponding energies (in parenthesis). 5

Figure S2. Correlation of calculated vs. experimental ^{77}Se NMR shifts for the main set of carbene Se adducts (**1**·Se - **24**·Se) (a - PBE0/6-311G(2d,2p)//PBE0/6-311+G(2d) level, b - PBE0/{6-311G(2d,2p);Se(NMR-DKH)}//PBE0/{6-311+G(2d);Se(NMR-DKH)}, c - PBE0/{6-311G(3df,2p);Se(NMR-DKH)}//PBE0/{6-311+G(3df,2p);Se(NMR-DKH)} level) 6

Figure S3. Correlation of calculated vs. experimental ^{77}Se NMR shifts for the main set of carbene Se adducts (**1**·Se - **24**·Se) (a - PBE/6-311G(2d,2p)//PBE0/6-311+G(2d) level, b - PBE50/6-311G(2d,2p)//PBE0/6-311+G(2d) level). 6

Table S3. Se-C bond lengths (L, Å) and some valence angles (°) for **1**•Se, **5**•Se, **7**•Se, **13**•Se, and **18**•Se calculated at DFT level of theory (PBE0) with different basis sets. 7

Table S4. Experimental (in $CDCl_3$) and calculated at non-relativistic and fully relativistic mDKS levels of theory ^{77}Se NMR shifts (ppm) for selected carbene·Se adducts (**1**·Se -**2**·Se, **4**·Se -**7**·Se, **13**·Se -**14**·Se, **17**·Se -**20**·Se). 8

Figure S4. Correlation of calculated vs. experimental ^{77}Se NMR shifts for the main set of carbene Se adducts (a - **1**·Se -**2**·Se, **4**·Se -**7**·Se, **13**·Se -**14**·Se, **17**·Se -**20**·Se, TZ_DZ//PBE0/6-311+G(2d) level, b - **1**·Se -**2**·Se, **6**·Se, **13**·Se -**14**·Se, **17**·Se -**19**·Se, TZ_DZ//PBE0/{6-311+G(3df,2p);Se(NMR-DKH)} level). 9

Table S5. Experimental (in $CDCl_3$) and calculated^a ^{77}Se NMR shifts (ppm) for some carbene·Se adducts (**13**·Se-**14**·Se, **17**·Se) and their associates with chloroform. 9

Table S6. Experimental (in $CDCl_3$ unless otherwise stated) and calculated ^{77}Se NMR shifts (ppm) for extended set of carbene·Se adducts (**1**·Se - **38**·Se). 10

Table S7. Experimental (in C_6D_6 unless otherwise stated) and calculated ^{31}P NMR shifts (ppm) for main set of carbene·PPh adducts (**3**·PPh, **5**·PPh, **11**·PPh, **13**·PPh, **26**·PPh-**29**·PPh, **35**·PPh, **39**·PPh-**40**·PPh). 12

Table S8. Experimental (in C_6D_6 unless otherwise stated) and calculated ^{31}P and ^{13}C NMR shifts (ppm) for extended set of carbene·PPh adducts (**3**·PPh, **5**·PPh, **11**·PPh, **13**·PPh, **25**·PPh-**29**·PPh, **35**·PPh, **37**·PPh, **39**·PPh-**44**·PPh). 13

Figure S5. Correlation of calculated (PBE0/6-311G(2d,2p)//PBE0/6-31+G(d) level) vs. experimental ^{31}P NMR shifts for extended set of carbene PPh adducts (**3**·PPh, **5**·PPh, **11**·PPh, **13**·PPh, **25**·PPh-**29**·PPh, **35**·PPh, **37**·PPh, **39**·PPh-**44**·PPh). 15

Table S9. Experimental (in C_6D_6) and calculated[a] ^{13}C NMR shifts (ppm) for set of carbenes (**3, 5, 11, 13, 25-29, 35, 39-44**). 16

Table S10. Experimental (in DMSO-d_6 unless otherwise stated) and calculated[a] ^{13}C NMR shifts (ppm) for set of carbene•H^+ azolium salts (**3**·H^+, **5**·H^+, **11**·H^+, **17**·H^+, **28**·H^+-**30**·H^+, **32**·H^+-**35**·H^+). 17

Figure S6. Structures of [$PdBr_2$(^{i}Pr_2-bimy)*L*] complexes. *L* = **3, 5, 11, 45-56.** 18

Table S11. Experimental (in $CDCl_3$) and calculated[a] ^{13}C NMR shifts (ppm) of ^{i}Pr_2-bimy carbenoid resonances in *trans*- [$PdBr_2$(^{i}Pr_2-bimy)*L*] complexes. 18

Table S1. Experimental (in $CDCl_3$) and calculated ^{77}Se NMR shifts (ppm) for main set of carbene·Se adducts (**1**·Se-**24**·Se).

№ of compound	Carbene	Exp. [a]	Calculated																
			BP86/TZPP//BP86/TZPP (PCM//PCM)[a,b]	PBE0/6-311G(2d,2p)//**PBE0**/ 6-31+G(d) **(vac //vac)**	**PBE0/6-311G(2d,2p)//PBE0/ 6-31+G(d) (vac //vac)**[d]	PBE0/6-311G(2d,2p)//PBE0/ 6-31+G(d) **(PCM//PCM)**	PBE0/6-311G(2d,2p)//PBE0/ 6-31+G(d) **(PCM//vac)**	PBE0/6-311G(2d,2p)//PBE0/ 6-31+G(d) **(vac//PCM)**	PBE0/6-311G(2d,2p)//PBE0/**6-311+G(2d)**	**PBE0/6-311G(2d,2p)//PBE0/6-311+G(2d)**[d]	PBE0/{6-311G(2d,2p);**Se(NMR-DKH)**}// PBE0/{6-311+G(2d);**Se(NMR-DKH)**}	PBE0/**6-311G(3df,2p)**//PBE0/6-311+G(2d)	PBE0/6-311G(3df,2p)//PBE0/**6-311+G(3df,2p)**	PBE0/{6-311G(3df,2p);**Se(NMR-DKH)**}// PBE0/{6-311+G(3df,2p);**Se(NMR-DKH)**}	**PBE0/{6-311G(3df,2p);Se(NMR-DKH)}// PBE0/{6-311+G(3df,2p);Se(NMR-DKH)}**[d]	**PBE**/6-311G(2d,2p)//PBE0/6-311+G(2d)	**PBE50**/6-311G(2d,2p)//PBE0/6-311+G(2d)	PBE0/6-311G(2d,2p)//**PBE**/6-311+G(2d)	**PBE**/6-311G(2d,2p)//**PBE**/6-311+G(2d)
	σ($SeMe_2$)[b]			1790		1814	1815	1791	1788		1693	1790	1793	1694		1699	1872	1742	1647
**1**	**ICy**	-22.1	1617.0	-74.1	**-39.0**	-113.9	-126.3	-52.3	-32.3	**-27.7**	-42.0	-21.3	-28.7	-45.6	**-30.2**	-56.7	-5.9	-37.6	-71.9
**2**	**IDD**	-11.3	1618.5	-60.3	**-27.5**	-102.8	-115.2	-42.9	-26.9	**-23.0**	-35.5	-16.7	-19.8	-30.2	**-18.3**	-50.3	-3.5	-28.4	-59.3
**3**	**IMes**	26.7	1535.7	-0.3	**22.3**	-42.5	-54.4	24.1	29.1	**25.8**	26.5	41.4	37.1	35.0	**32.2**	10.1	54.5	40.1	10.1
**4**	**SIDD**	75.0	1521.3	44.9	**59.9**	-14.8	-25.4	61.7	74.2	**65.1**	69.2	80.7	79.0	73.8	**62.3**	52.1	94.9	72.8	42.7
**5**	**SIMes**	109.7	1462.6	125.0	**126.4**	61.3	57.2	141.8	124.6	**109.0**	127.0	131.9	130.3	126.6	**103.2**	97.9	151.8	138.6	101
**6**	**I^{i}Pr^{Me}**	-18.2	1608.0	-46.3	**-15.9**	-85.8	-101.8	-23	-22.6	**-19.2**	-34.7	-11.1	-13.5	-29.2	**-17.5**	-49.9	6.0	-24.6	-59.3
**7**	**IMe**	29.9	1531.1	4.5	**26.3**	-37.3	-48.7	28.4	32.5	**28.8**	30.6	44.5	40.4	28.9	**27.5**	13.8	57.6	43.5	13.9
**8**	**IPent**	101.2	1427.2	119.8	**122.1**	99.3	93.5	138.4	131.9	**115.4**	134.6	141.2	139.2	135.4	**110.1**	126.0	141.6	134.7	120.6
**9**	**INon**	101.9	1427.5	128.6	**129.4**	105.7	103.8	136.8	128.5	**112.4**	134.4	139.4	137.2	135.1	**109.8**	122.0	138.7	131.5	116.8
**10**	**IHept**	99.8	1435.9	124.8	**126.3**	98.6	95.4	133.8	128.5	**112.4**	134.1	139.5	137.3	134.9	**109.7**	122.3	138.5	131.4	117
**11**	**IPr**	90.0	1441.9	90.0	**97.4**	43.3	41.6	103.2	115.1	**100.8**	130.5	124.7	122.5	133.4	**108.5**	112.9	123.5	119.6	109.1
**12**	**IPr^{OMe}**	86.5	1443.6	88.4	**96.0**	37.5	34	102.6	116.5	**102.0**	131.2	125.8	123.7	133.9	**108.9**	113.1	125.4	121.3	109.7
**13**	**SIPr**	190.0	1347.2	209.0	**196.2**	152.9	153	215.6	228.5	**199.6**	244.2	231.8	229.7	245.5	**195.4**	217.2	235.9	231.4	214.5

**14**	**SIPr^{OMe}**	184.6	1349.5	194.2	**183.9**	144.7	136.8	205.2	220.4	**192.5**	236.7	225.7	224.1	238.0	**189.6**	210.3	231.1	224.5	206.4
**15**	**IPr***	106.0	1442.9	89.7	**97.1**	85.8	82.1	100.6	107.2	**93.9**	120.6	118.6	116.5	124.6	**101.7**	112.5	111.3	107.8	105.1
**16**	**IPr*^{OMe}**	104.0	1439.1	93.5	**100.3**	90.3	86	104.8	112.4	**98.4**	124.2	124.4	122.1	128.4	**104.6**	117.7	116.3	113.6	110.7
**17**	**IPr^{Cl}**	173.6	1347.4	177.0	**169.7**	146.3	139.5	188.8	199.9	**174.7**	214.3	207.9	205.8	218.0	**174.1**	204.7	198.9	203.4	202.5
**18**	**I^{t}Bu**	182.6	1354.9	167.0	**161.3**	133.4	120.4	173.4	189.3	**165.4**	202.0	195.9	195.0	214.2	**171.1**	170.1	196.8	193	172.2
**19**	**IAd**	196.9	1360.6	176.4	**169.2**	146.9	136.3	180.9	192.3	**168.0**	207.7	201.6	199.9	217.3	**173.5**	178.9	204.8	202	182.3
**20**	**Tr1**	33.5	1498.3	17.8	**37.4**	-33.1	-46.2	32.6	41.8	**36.9**	37.6	47.7	44.1	37.1	**33.9**	21.6	59.7	52.7	25.2
**21**	**Tr2**	76.1	1441.8	78.7	**88.0**	21.7	8.3	92.8	99.4	**87.1**	91.4	102.8	99.5	90.5	**75.3**	79.4	115.1	115.2	88.9
**22**	**Tr3**	73.6	1473.5	73.3	**83.5**	16.5	4	86.2	92.3	**80.9**	86.9	95.9	93.6	88.0	**73.3**	80.0	101.8	96.3	76.9
**23**	**Tr4**	46.9	1508.4	12.9	**33.3**	-13.9	-24.2	24.5	33.1	**29.3**	33.3	39.5	35.6	33.8	**31.3**	7.4	54.2	40.5	7.7
**24**	**Tr5**	131.2	1304.5	122.6	**-39.0**	72.6	77.6	121.6	159.3	**-27.7**	165.7	165.6	168.6	171.8	**-30.2**	152.2	168.4	184.7	171.1
	R^2		*0.887 (0.938[c])*	*0.951*	***0.951***	*0.923*	*0.913*	*0.940*	*0.969*	***0.969***	*0.969*	*0.969*	*0.968*	*0.975*	***0.975***	*0.949*	*0.967*	*0.958*	*0.948*
	RMSE				***14.7***					***11.5***					***10.3***				

[a] data from the ref. [20]; [b] shielding; [c] evaluated without **Tr5**; [c]scaled.

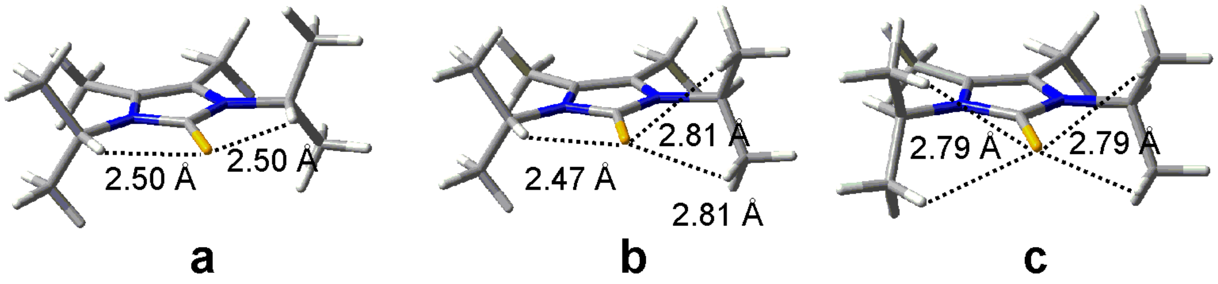

Figure S1. Different forms of **I*^i*Pr^Me**·Se (**6**·Se) with corresponding distances (Å) between *^i*Pr hydrogen's and Se.

Table S2. ^{77}Se NMR shifts (ppm) for **ICy** (**1** Se), **IDD** (**2** Se) and **I^iPr^Me** (**6** Se) Se-adducts. Experimental (in $CDCl_3$) and calculated[a] CSs for different forms with corresponding energies (in parenthesis).

№ of compound	Carbene	Exp.	Calculated NMR shifts (Energy), ppm (kcal/mol)		
			**a**[b]	**b**[b]	**c**[b]
**1**	**ICy**	-22.1	-39.0 (0.0)	40.4 (1.5)	99.2 (4.2)
**2**	**IDD**	-11.3	-27.5 (0.0)	30.9 (1.6)	80.2 (3.9)
**6**	**I*^i*Pr^Me**	-18.2	-15.9 (0.0)	46.5 (0.3)	109.8 (1.5)

[a] at PBE0/6-311G(2d,2p)//PBE0/6-31+G(d) level and scaled; [b] structure of isomers as on Figure S1.

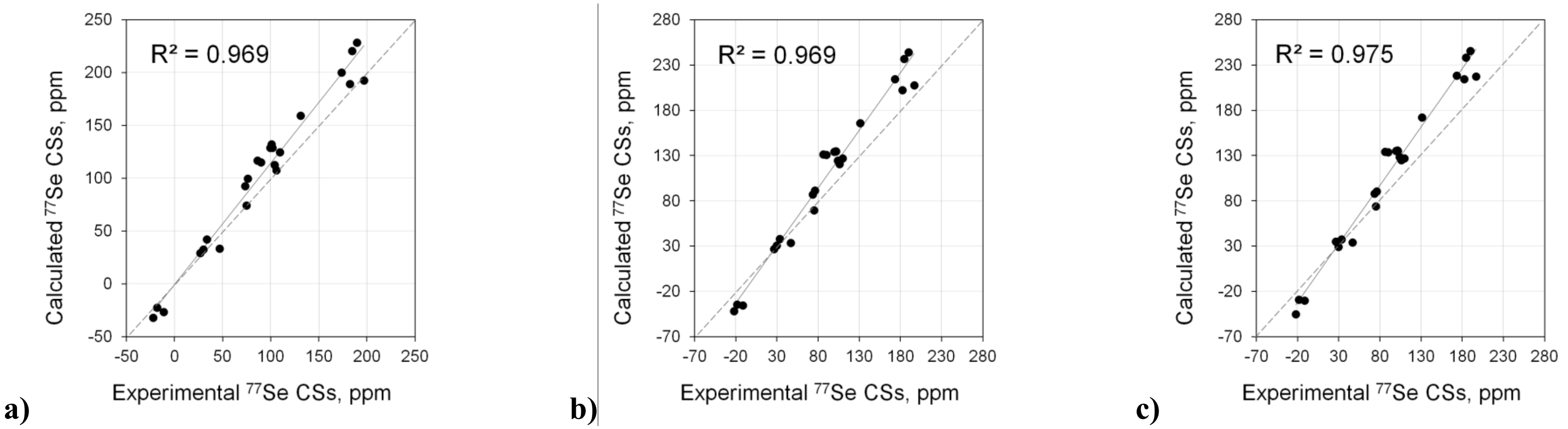

Figure S2. Correlation of calculated vs. experimental ^{77}Se NMR shifts for the main set of carbene Se adducts (**1**·Se - **24**·Se) (a - PBE0/6-311G(2d,2p)//PBE0/6-311+G(2d) level, b - PBE0/{6-311G(2d,2p);Se(NMR-DKH)}//PBE0/{6-311+G(2d);Se(NMR-DKH)}, c - PBE0/{6-311G(3df,2p);Se(NMR-DKH)}//PBE0/{6-311+G(3df,2p);Se(NMR-DKH)} level).

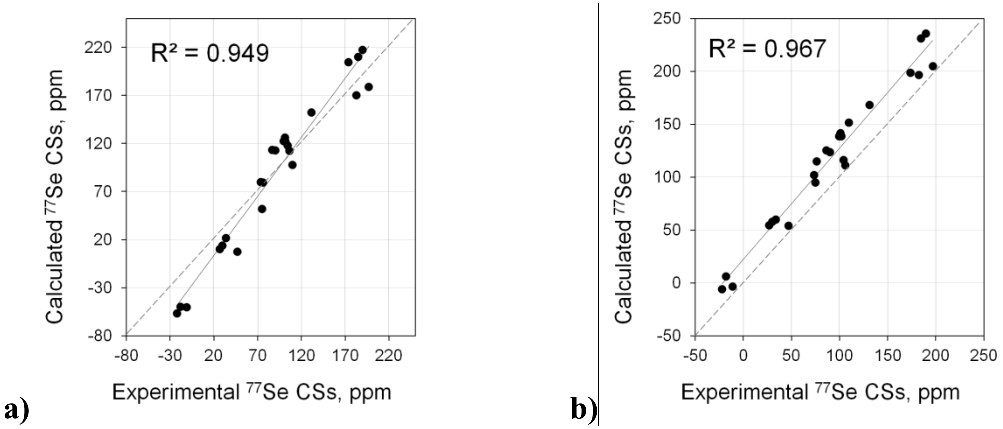

Figure S3. Correlation of calculated vs. experimental ^{77}Se NMR shifts for the main set of carbene Se adducts (**1**·Se - **24**·Se) (a - PBE/6-311G(2d,2p)//PBE0/6-311+G(2d) level, b - PBE50/6-311G(2d,2p)//PBE0/6-311+G(2d) level).

Table S3. Se-C bond lengths (L, Å) and some valence angles (°) for 1•Se, 5•Se, 7•Se, 13•Se, and 18•Se calculated at DFT level of theory (PBE0) with different basis sets.

Carbene	Basis sets	L (Se-C^{Carbene})	∠N-C^{Carbene}-Se	∠N-C ^{Carbene} -N
**1**	6-31+G(d)	*1.811*	*127.31*	*105.38*
**ICy**	6-311+G(2d)	1.835	127.26	105.47
	6-311+G(3df,2p)	1.832	127.30	105.39
	6-311+G(2d) Se - NMR_DKH	1.834	127.28	105.44
	6-311+G(3df,2p) Se - NMR_DKH	1.833	127.32	105.35
**5**	6-31+G(d)	*1.795*	*125.82*	*108.37*
**SIMes**	6-311+G(2d)	1.818	125.73	108.53
	6-311+G(3df,2p)	1.816	125.76	108.49
	6-311+G(2d) Se - NMR_DKH	1.817	125.76	108.48
	6-311+G(3df,2p) Se - NMR_DKH	1.816	125.76	108.48
**7**	6-31+G(d)	*1.800*	*127.50*	*104.99*
**IMe**	6-311+G(2d)	1.821	127.57	104.87
	6-311+G(3df,2p)	1.818	127.58	104.84
	6-311+G(2d) Se - NMR_DKH	1.820	127.59	104.82
	6-311+G(3df,2p) Se - NMR_DKH	1.819	127.58	104.83
**13**	6-31+G(d)	*1.796*	*126.37*	*108.23*
**SIPr**	6-311+G(2d)	1.817	125.81	108.38
	6-311+G(3df,2p)	1.815	125.83	108.35
	6-311+G(2d) Se - NMR_DKH	1.816	125.83	108.34
	6-311+G(3df,2p) Se - NMR_DKH	1.815	125.83	108.35
**18**	6-31+G(d)	*1.827*	*127.09*	*105.82*
**I^{t}Bu**	6-311+G(2d)	1.847	127.13	105.74
	6-311+G(3df,2p)	1.844	127.14	105.72
	6-311+G(2d) Se - NMR_DKH	1.845	127.14	105.70
	6-311+G(3df,2p) Se - NMR_DKH	1.845	127.14	105.72

Table S4. Experimental (in $CDCl_3$) and calculated at non-relativistic and fully relativistic mDKS levels of theory ^{77}Se NMR shifts (ppm) for selected carbene·Se adducts (**1**·Se -**2**·Se, **4**·Se -**7**·Se, **13**·Se -**14**·Se, **17**·Se -**20**·Se).

№ of compound	Carbene	Exp.	Calculated				
			non-relativistic		relativistic		
			PBE0/6-311G(2d,2p)// PBE0/6-311+G(2d)	PBE0/{6-311G(3df,2p); Se(NMR-DKH)}// PBE0/{6-311+G(3df,2p); Se(NMR-DKH)}	"TZ_DZ"[b]// PBE0/6-311+G(2d)	"DTZ_TZ_DZ"[b]// PBE0/6-311+G(2d)	"TZ_DZ"[b]// PBE0/{6-311+G(3df,2p); Se(NMR-DKH)}
	σ($SeMe_2$)[a]		1788	1694	2066	2034	2070
**1**	**ICy**	-22.1	-32.3	-45.6	-5.4	-40.5	-11.4
**2**	**IDD**	-11.3	-26.9	-30.2	-3.9	-36.9	-3.6
**4**	**SIDD**	75.0	74.2	73.8	102.3		
**5**	**SIMes**	109.7	124.6	126.6	158.9	124.4	
**6**	**I*i*Pr^{Me}**	-18.2	-22.6	-29.2	4.4	-30.0	4.5
**7**	**IMe**	29.9	32.5	28.9	65.1		
**13**	**SIPr**	190.0	228.5	245.5	250.7	233.1	250.4
**14**	**SIPr^{OMe}**	184.6	220.4	238.0	244.4	227.6	244.1
**17**	**IPr^{Cl}**	173.6	199.9	218.0	218.3	202.2	215.9
**18**	**I*t*Bu**	182.6	189.3	214.2	215.4	191.7	215.8
**19**	**IAd**	196.9	192.3	217.3	226.4	199.3	208.8
**20**	**Tr1**	33.5	41.8	37.1	75.9		

[a] shielding; [b] "TZ_DZ" - 4c-mDKS: PBE0/{ucc-pvtz+ucc-pvdz, Se(dyall-v**d**z)}, "DTZ_TZ_DZ" - 4c-mDKS: PBE0/{ucc-pvtz+ucc-pvdz,Se(dyall-v**t**z)}.

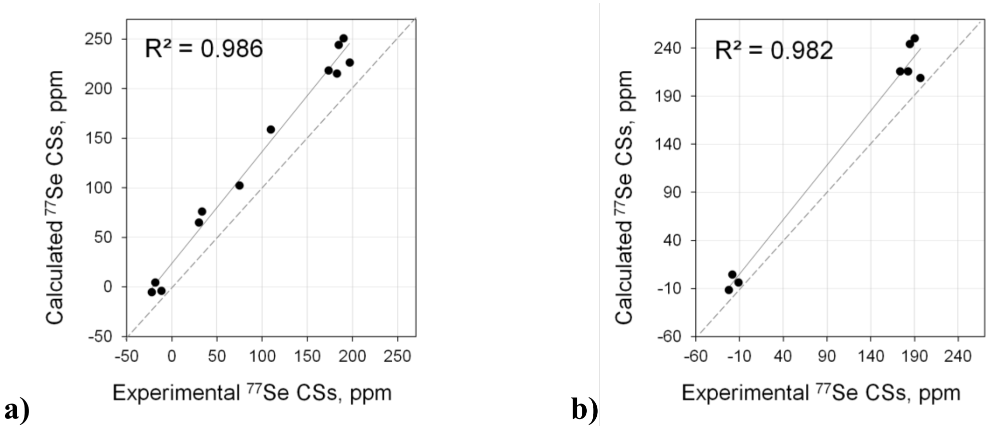

Figure S4. Correlation of calculated vs. experimental ^{77}Se NMR shifts for the main set of carbene Se adducts (a - **1**·Se **-2**·Se**, 4**·Se **-7**·Se**, 13**·Se **-14**·Se**, 17**·Se **-20**·Se, TZ_DZ//PBE0/6-311+G(2d) level, b - **1**·Se **-2**·Se**, 6**·Se**, 13**·Se **-14**·Se**, 17**·Se **-19**·Se, TZ_DZ//PBE0/{6-311+G(3df,2p);Se(NMR-DKH)} level).

Table S5. Experimental (in $CDCl_3$) and calculated[a] ^{77}Se NMR shifts (ppm) for some carbene·Se adducts (**13**·Se-**14**·Se**, 17**·Se) and their associates with chloroform.

№ of compound	Carbene	Exp.	Calc.	
			NHC-Se	NHC-Se***$CHCl_3$**
**13**	**SIPr**	190.0	199.6	178.4
**14**	**SIPr^{OMe}**	184.6	192.5	172.7
**17**	**IPr^{Cl}**	173.6	174.7	161.1

[a] at PBE0/6-311G(2d,2p)//PBE0/6-311+G(2d) level and scaled;

Table S6. Experimental (in $CDCl_3$ unless otherwise stated) and calculated ^{77}Se NMR shifts (ppm) for extended set of carbene·Se adducts (**1·Se - 38·Se**).

№ of compound	Carbene	Exp.[a]	Calculated					
			PBE0/6-311G(2d,2p)// PBE0/6-31+G(d)	**PBE0/6-311G(2d,2p)// PBE0/6-31+G(d)**[b]	PBE0/6-311G(2d,2p)// PBE0/6-311+G(2d)	**PBE0/6-311G(2d,2p)// PBE0/6-311+G(2d)**[b]	PBE0/{6-311G(3df,2p); Se(NMR-DKH)}// PBE0/{6-311+G(3df,2p); Se(NMR-DKH)}	**PBE0/{6-311G(3df,2p); Se(NMR-DKH)}// PBE0/{6-311+G(3df,2p); Se(NMR-DKH)}**[b]
**1**	**ICy**	-22.1	-74.1	**-48.8**	-32.3	**-33.7**	-45.6	**-42.6**
**2**	**IDD**	-11.3	-60.3	**-36.5**	-26.9	**-28.7**	-30.2	**-29.2**
**3**	**IMes**	26.7	-0.3	**17.2**	29.1	**22.4**	35.0	**27.2**
**4**	**SIDD**	75.0	44.9	**57.7**	74.2	**63.5**	73.8	**60.9**
**5**	**SIMes**	109.7	125.0	**129.3**	124.6	**109.5**	126.6	**106.6**
**6**	**I^iPr^{Me}**	-18.2	-46.3	**-24.0**	-22.6	**-24.8**	-29.2	**-28.4**
**7**	**IMe**	29.9	4.5	**21.5**	32.5	**25.5**	28.9	**22.0**
**8**	**IPent**	101.2	119.8	**124.7**	131.9	**116.2**	135.4	**114.2**
**9**	**INon**	101.9	128.6	**132.6**	128.5	**113.1**	135.1	**114.0**
**10**	**IHept**	99.8	124.8	**129.2**	128.5	**113.1**	134.9	**113.8**
**11**	**IPr**	90.0	90.0	**98.0**	115.1	**100.8**	133.4	**112.5**
**12**	**IPr^{OMe}**	86.5	88.4	**96.6**	116.5	**102.1**	133.9	**112.9**
**13**	**SIPr**	190.0	209.0	**204.5**	228.5	**204.3**	245.5	**209.6**
**14**	**$SIPr^{OMe}$**	184.6	194.2	**191.3**	220.4	**196.9**	238.0	**203.1**
**15**	**IPr***	106.0	89.7	**97.8**	107.2	**93.6**	124.6	**104.9**
**16**	**$IPr*^{OMe}$**	104.0	93.5	**101.2**	112.4	**98.4**	128.4	**108.2**
**17**	**IPr^{Cl}**	173.6	177.0	**175.9**	199.9	**178.2**	218.0	**185.8**
**18**	**I^tBu**	182.6	167.0	**166.9**	189.3	**168.5**	214.2	**182.5**

**19**	**IAd**	196.9	176.4	**175.3**	192.3	**171.3**	217.3	**185.2**
**20**	**Tr1**	33.5	17.8	**33.4**	41.8	**33.9**	37.1	**29.1**
**21**	**Tr2**	76.1	78.7	**87.9**	99.4	**86.5**	90.5	**75.3**
**22**	**Tr3**	73.6	73.3	**83.1**	92.3	**80.0**	88.0	**73.2**
**23**	**Tr4**	46.9	12.9	**29.0**	33.1	**26.0**	33.8	**26.2**
**24**	**Tr5**	131.2	122.6	**127.2**	159.3	**141.2**	171.8	**145.8**
**25**		593.0[c]	646.9	**596.4**	649.0	**588.0**	678.2	**584.5**
**26**	**ITME**	3.0[c]	-46.4	**-24.0**	-17.1	**-19.8**	-22.0	**-22.1**
**27**	**I^{i}Pr**	-3.0[c]	-56.4	**-33.0**	-22.4	**-24.6**	-42.0	**-39.5**
**28**	**ThIPr**	396.0[c]	513.0	**476.6**	504.1	**455.8**	531.1	**457.0**
**29**	**BI^{i}Pr**	67.0[c]	56.8	**68.3**	76.9	**66.0**	74.8	**61.7**
**30**	**AAC1**	437.0[c]	525.5	**487.8**	546.7	**494.6**	573.7	**493.9**
**31**		183.0[c]	207.9	**203.5**	222.1	**198.5**	232.3	**198.2**
**32** (C_a)		516.0	542.9	**503.3**	564.4	**510.8**	585.2	**503.9**
**32** (C_b)		113.0	127.9	**131.9**	150.9	**133.5**	155.1	**131.3**
**33**	**6-Mes**	271.0[c]	280.0	**268.1**	292.9	**263.1**	291.8	**249.7**
**34**	**Trazy**	196.0[c]	167.1	**167.0**	206.5	**184.2**	213.6	**182.0**
**35**	**CAC-Mes**	472.0[c]	522.2	**484.8**	532.3	**481.5**	553.5	**476.4**
**36**	**6-CAAC**	1180.0[c]	1288.5	**1170.6**	1288.6	**1171.5**	1360.8	**1175.8**
**37**	**DAC1a**	847.0[c]	874.8	**800.4**	894.3	**811.8**	934.3	**806.3**
**38**	**^{i}Pr$_2$-pery**	364.0[c]	349.9	**330.6**	366.8	**330.5**	386.5	**331.8**
	R^2		*0.991*	***0.991***	*0.994*	***0.994***	*0.993*	***0.993***
	RMSE			***23.8***		***19.3***		***21.0***

[a] data from the ref. [11,18, 20]; [b]scaled; [c]in acetone-d_6.

Table S7. Experimental (in C_6D_6 unless otherwise stated) and calculated ^{31}P NMR shifts (ppm) for main set of carbene·PPh adducts (**3**·PPh, **5**·PPh, **11**·PPh, **13**·PPh, **26**·PPh-**29**·PPh, **35**·PPh, **39**·PPh-**40**·PPh).

№ of compound	Carbene	Exp.[a]	Calculated						
			BP86/TZPP// BP86/TZPP[a b]	PBE0/6-311G(2d,2p)// PBE0/6-31+G(d)	**PBE0/6-311G(2d,2p)// PBE0/6-31+G(d)**[c]	PBE0/6-311G(2d,2p)// PBE0/6-311+G(2d)	**PBE0/6-311G(2d,2p)// PBE0/6-311+G(2d)**[c]	PBE0/6-311G(3df,2p)// PBE0/6-311+G(2d)	**PBE0/6-311G(3df,2p)// PBE0/6-311+G(2d)**[c]
**3**	**IMes**	-23.0	402.1	-35.0	**-25.6**	-28.9	**-26.1**	-30.4	**-26.2**
**5**	**SIMes**	-10.4	387.3	-18.1	**-9.6**	-12.4	**-10.4**	-14.4	**-11.2**
**11**	**IPr**	-18.9	389.6	-27.1	**-18.1**	-20.1	**-17.7**	-21.5	**-17.9**
**13**	**SIPr**	-10.2[d]	383.1	-15.4	**-7.0**	-8.6	**-6.7**	-10.7	**-7.7**
**26**	**ITME**	-53.5[e]	424.0	-64.7	**-53.8**	-56.9	**-52.7**	-58.8	**-52.9**
**27**	**I^{i}Pr**	-61.2	430.2	-73.1	**-61.8**	-67.0	**-62.4**	-68.6	**-62.1**
**28**	**ThIPr**	57.0	335.3	52.4	**57.3**	59.0	**57.6**	59.6	**58.2**
**29**	**BI^{i}Pr**	-34.6	408.2	-44.8	**-34.9**	-37.8	**-34.6**	-39.4	**-34.7**
**35**	**CAC-Mes**	39.7	337.1	32.0	**37.9**	39.0	**38.6**	37.1	**37.1**
**39**	**CAAC-IPr**	68.9	321.5	64.2	**68.5**	70.4	**68.5**	69.8	**67.8**
**40**	**6-IPr**	14.8	359.7	8.7	**15.8**	13.7	**14.5**	16.9	**18.2**
	R^2		*0.987*	*0.999*	***0.999***	*0.999*	***0.999***	*0.998*	***0.998***
					1.5		***1.6***		***1.9***

[a] data from the ref. [20]; [b] shielding; [c]scaled; [d] in $CDCl_3$; [e] in THF-d_8

Table S8. Experimental (in C_6D_6 unless otherwise stated) and calculated ^{31}P and ^{13}C NMR shifts (ppm) for extended set of carbene·PPh adducts (**3**·PPh, **5**·PPh, **11**·PPh, **13**·PPh, **25**·PPh-**29**·PPh, **35**·PPh, **37**·PPh, **39**·PPh-**44**·PPh).

№ of compound	Carbene	δ(^{31}P)							δ(^{13}C)						
		Exp.[a]	Calculated						Exp.[a]	Calculated					
			PBE0/6-311G(2d,2p)//PBE0/6-31+G(d)	**PBE0/6-311G(2d,2p)//PBE0/6-31+G(d)**[b]	PBE0/6-311G(2d,2p)//PBE0/6-311+G(2d)	**PBE0/6-311G(2d,2p)//PBE0/6-311+G(2d)**[b]	PBE0/6-311G(3df,2p)//PBE0/6-311+G(2d)	**PBE0/6-311G(3df,2p)//PBE0/6-311+G(2d)**[b]		PBE0/6-311G(2d,2p)//PBE0/6-31+G(d)	**PBE0/6-311G(2d,2p)//PBE0/6-31+G(d)**[b]	PBE0/6-311G(2d,2p)//PBE0/6-311+G(2d)	**PBE0/6-311G(2d,2p)//PBE0/6-311+G(2d)**[b]	PBE0/6-311G(3df,2p)//PBE0/6-311+G(2d)	**PBE0/6-311G(3df,2p)//PBE0/6-311+G(2d)**[b]
**3**	**IMes**	-23.0	-35.0	**-24.4**	-28.9	**-24.9**	-30.4	**-25.1**	170.0[d]	170.2	**165.8**	169.8	**165.9**	172.0	**166.0**
**5**	**SIMes**	-10.4	-18.1	**-9.1**	-12.4	**-9.9**	-14.4	**-10.5**	184.3[d]	186.9	**180.5**	186.3	**180.7**	188.3	**180.7**
**11**	**IPr**	-18.9	-27.1	**-17.2**	-20.1	**-16.9**	-21.5	**-17.0**	172.9	175.8	**170.7**	175.3	**170.8**	177.4	**170.9**
**13**	**SIPr**	-10.2[c]	-15.4	**-6.7**	-8.6	**-6.4**	-10.7	**-7.2**	186.9[c]	193.9	**186.7**	192.7	**186.5**	194.6	**186.4**
**25**		69.5	65.7	**66.4**	72.2	**67.5**	71.3	**67.4**	199.7	207.0	**198.3**	206.4	**198.8**	208.3	**198.7**
**26**	**ITME**	-53.5[d]	-64.7	**-51.1**	-56.9	**-50.6**	-58.8	**-50.9**	169.1[d]	174.9	**169.9**	174.6	**170.2**	177.0	**170.5**
**27**	**I^{i}Pr**	-61.2	-73.1	**-58.7**	-67.0	**-59.8**	-68.6	**-59.8**	167.8	174.4	**169.5**	173.8	**169.5**	176.5	**170.1**
**28**	**ThIPr**	57.0	52.4	**54.4**	59.0	**55.5**	59.6	**56.7**	192.0	204.0	**195.7**	203.5	**196.2**	204.7	**195.4**
**29**	**BI^{i}Pr**	-34.6	-44.8	**-33.2**	-37.8	**-33.1**	-39.4	**-33.3**	177.4	181.6	**175.9**	181.2	**176.1**	183.6	**176.5**
**35**	**CAC-Mes**	39.7	32.0	**36.0**	39.0	**37.2**	37.1	**36.3**	180.3	186.6	**180.3**	184.8	**179.4**	186.6	**179.2**
**37**	**DAC1a**	83.0	75.4	**75.1**	81.1	**75.7**	80.0	**75.3**	172.0	182.4	**176.6**	181.5	**176.4**	183.4	**176.3**
**39**	**CAAC-IPr**	68.9	64.2	**65.0**	70.4	**65.9**	69.8	**66.0**	208.1	218.0	**208.1**	217.2	**208.5**	219.1	**208.4**
**40**	**6-IPr**	14.8	8.7	**15.0**	13.7	**14.0**	16.9	**17.9**	186.5	193.6	**186.5**	192.1	**186.0**	195.4	**187.1**
**41**	**6-NHC^{pyr}**	34.9	38.1	**41.5**	44.0	**41.7**	42.5	**41.2**	199.9	208.2	**199.4**	207.7	**200.0**	209.7	**199.9**
**42**	**CAAC^{Menthyl}**	56.2	50.2	**52.4**	55.5	**52.3**	53.9	**51.5**	191.3	200.1	**192.2**	199.0	**192.1**	200.5	**191.7**
**43**		126.3[c]	146.2	**138.9**	147.4	**136.3**	146.8	**136.0**	217.2	229.7	**218.4**	227.2	**217.5**	229.3	**217.6**

**44**		-34.9	-53.1	**-40.7**	-46.3	**-40.9**	-47.7	**-40.8**	138.5	140.5	**139.5**	140.2	**139.3**	141.6	**138.7**
	R^2		*0.992*	***0.992***	*0.993*	***0.993***	*0.993*	***0.993***		*0.985*	***0.985***	*0.985*	***0.985***	*0.986*	***0.986***
	RMSE			***4.8***		***4.3***		***4.3***			***2.2***		***2.2***		***2.1***

[a] data from the ref. [20-21]; [b]scaled; [c]in $CDCl_3$; [d] in THF-d_8.

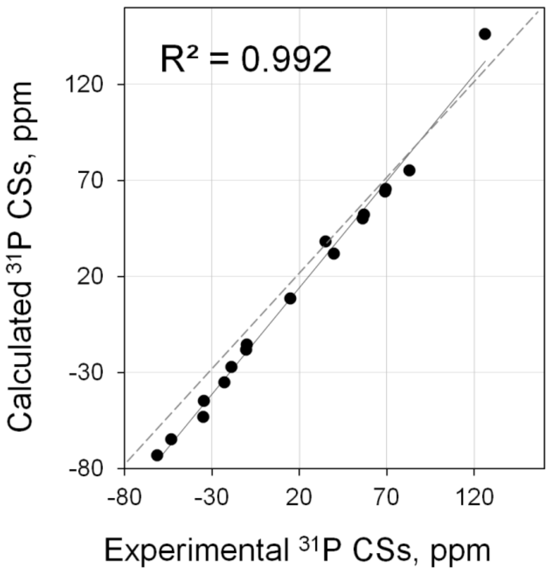

Figure S5. Correlation of calculated (PBE0/6-311G(2d,2p)//PBE0/6-31+G(d) level) vs. experimental ^{31}P NMR shifts for extended set of carbene PPh adducts (**3**·PPh, **5**·PPh, **11**·PPh, **13**·PPh, **25**·PPh-**29**·PPh, **35**·PPh, **37**·PPh, **39**·PPh-**44**·PPh).

Table S9. Experimental (in C_6D_6) and calculated[a] ^{13}C NMR shifts (ppm) for set of carbenes (**3, 5, 11, 13, 25-29, 35, 39-44**).

№ of compound	Carbene	Exp.[b]	Calc.	
				scaled
**3**	**IMes**	219.7	228.7	**218.5**
**5**	**SIMes**	243.8	258.0	**242.8**
**11**	**IPr**	220.6	229.7	**219.3**
**13**	**SIPr**	244.0	255.4	**240.6**
**25**		255.5	268.7	**251.7**
**26**	**ITME**	213.7	224.3	**214.8**
**27**	**I^{i}Pr**	210.5	222.4	**213.3**
**28**	**ThIPr**	254.4	269.4	**252.2**
**29**	**BI^{i}Pr**	221.6	238.6	**226.7**
**35**	**CAC-Mes**	260.56	279.0	**260.2**
**39**	**CAAC-IPr**	309.4	344.1	**314.2**
**40**	**6-Ipr**	245.1	256.6	**241.6**
**41**	**6-NHC^{pyr}**	282.0	301.7	**279.0**
**42**	**CAAC^{Menthyl}**	319.0	348.3	**317.6**
**43**		330.5	368.8	**334.6**
**44**		185.1	192.4	**188.4**
	R^2		*0.994*	***0.994***
	RMSE			***3.0***

[a] at PBE0/6-311G(2d,2p)//PBE0/6-31+G(d) level; [b] data from the ref. [21].

Table S10. Experimental (in DMSO-d_6 unless otherwise stated) and calculated[a] ^{13}C NMR shifts (ppm) for set of carbene•H^+ azolium salts (**3**·H^+, **5**·H^+, **11**·H^+, **17**·H^+, **28**·H^+-**30**·H^+, **32**·H^+-**35**·H^+).

№ of compound	Carbene	Exp.[b]	Calc.	
				scaled
**3**	**IMes**	139.9[b]	139.3	**139.1**
**5**	**SIMes**	160.2	164.3	**162.0**
**11**	**IPr**	139.4	140.4	**140.1**
**17**	**IPr^{Cl}**	138.4	136.8	**136.8**
**28**	**ThIPr**	158.5	158.3	**156.5**
**29**	**BI^{i}Pr**	140.0	137.9	**137.8**
**30**	**AAC1**	146.3	151.0	**149.8**
**32** (C_a)		155.4	151.1	**149.9**
**32** (C_b)		144.4	149.6	**148.5**
**33**	**6-Mes**	155.6	161.2	**159.1**
**34**	**Trazy**	154.2	151.7	**150.5**
**35**	**CAC-Mes**	160.4[b]	164.8	**162.4**
	R^2		*0.890*	***0.890***
	RMSE			***3.0***

[a] at PBE0/6-311G(2d,2p)//PBE0/6-31+G(d) level; [b] in $CDCl_3$; [b] data from the ref. [18].

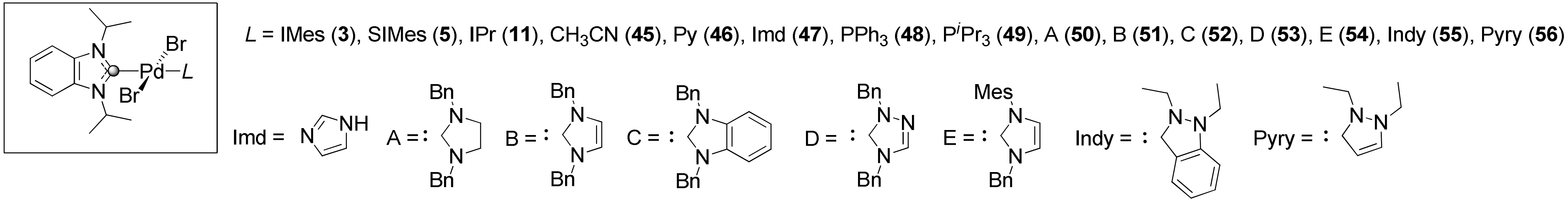

Figure S6. Structures of [PdBr$_2$(^{i}Pr$_2$-bimy)*L*] complexes. *L* = **3**, **5**, **11**, **45-56**.

Table S11. Experimental (in CDCl$_3$) and calculated[a] ^{13}C NMR shifts (ppm) of ^{i}Pr$_2$-bimy carbenoid resonances in *trans*- [PdBr$_2$(^{i}Pr$_2$-bimy)*L*] complexes.

№ of compound	Carbene (*L*)	Exp.[b]	Calc.	
				scaled
**3**	**IMes**	177.2	187.4	**160.1**
**5**	**SIMes**	177.6	188.2	**161.3**
**11**	**IPr**	177.5	188.5	**160.1**
**45**	**CH_3CN**	158.4	173.7	**172.1**
**46**	**Py**	160.0	173.7	**172.2**
**47**	**Imd**	161.4	174.8	**177.4**
**48**	**PPh_3**	173.1	184.5	**179.5**
**49**	**P^{i}Pr$_3$**	175.9	184.6	**183.2**
**50**	**A**	180.1	193.2	**184.4**
**51**	**B**	179.0	191.2	**181.8**
**52**	**C**	178.3	191.0	**179.2**
**53**	**D**	176.6	189.3	**178.2**
**54**	**E**	178.3	190.1	**175.3**
**55**	**Indy**	181.6	194.5	**176.1**
**56**	**Pyry**	182.4	195.6	**176.5**
	R^2		*0.961*	***0.961***
	RMSE			***1.6***

[a]4c-mDKS:PBE0/{ucc-pvtz+ucc-pvdz;Pd(dyall-vdz)}//KS:PBE0/{6-31+G(d);Pd(SDD)} level; [b] data from the ref. [15-16].
